# Developing a minimally invasive gene therapy for multiple sclerosis

**DOI:** 10.1016/j.omtm.2025.101543

**Published:** 2025-08-13

**Authors:** Paul J.H. Nijhuis, Maurits Romijn, Roy Honing, Giselle van Zon, Inge Huitinga, Fred de Winter, Joost Verhaagen

## Main text

(Molecular Therapy: Methods & Clinical Development 33, 1–22; September 2025)

In the originally published version of this article, in Figure 2A, the representative images for hSyn1-eGFP co-localization with GFAP and GS were inadvertently interchanged by the authors. In Figure 2B, the gfa2 brain stem image showing eGFP and Olig2 co-localization was mistakenly selected from the gfaABC1D group. This has now been corrected online.

**Figure:**
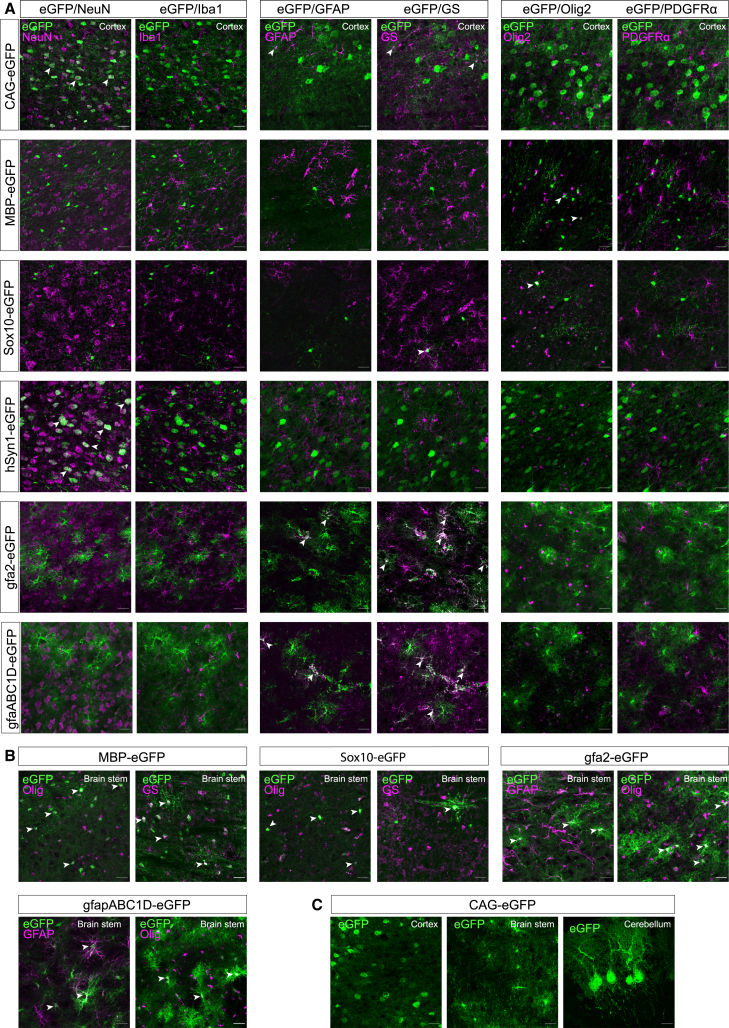
Figure 2. AAV-PHP.eB-mediated delivery of neural promoter constructs directs cell-type-specific transgene expression in the brain (original)

**Figure:**
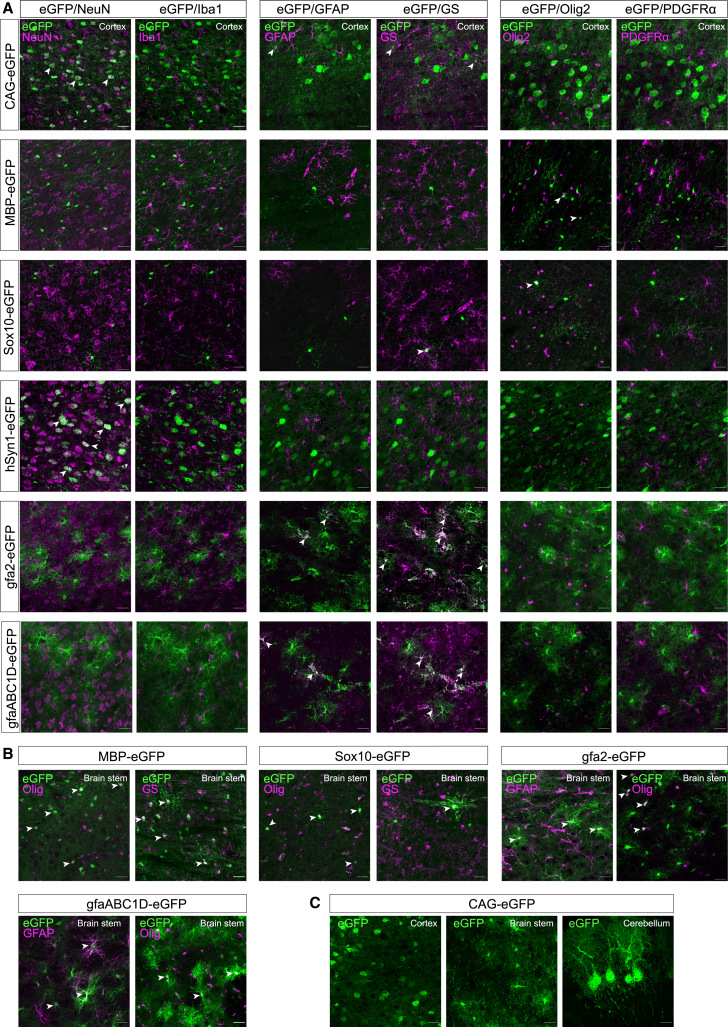
Figure 2. AAV-PHP.eB-mediated delivery of neural promoter constructs directs cell-type-specific transgene expression in the brain (corrected)

