# Supplementary material for: Developing a minimally invasive gene therapy for multiple sclerosis
Source: Mol Ther Methods Clin Dev. 2025 Jun 10;33(3):101504. doi: 10.1016/j.omtm.2025.101504 (PMC12536318; doi:10.1016/j.omtm.2025.101504)
Supplement: Document S1. Figures S1–S7 [file mmc1.pdf]

OMTM, Volume *33*

Supplemental information

Developing a minimally invasive gene therapy for multiple sclerosis

Paul J.H. Nijhuis, Maurits Romijn, Roy Honing, Giselle van Zon, Inge Huitinga, Fred de Winter, and Joost Verhaagen

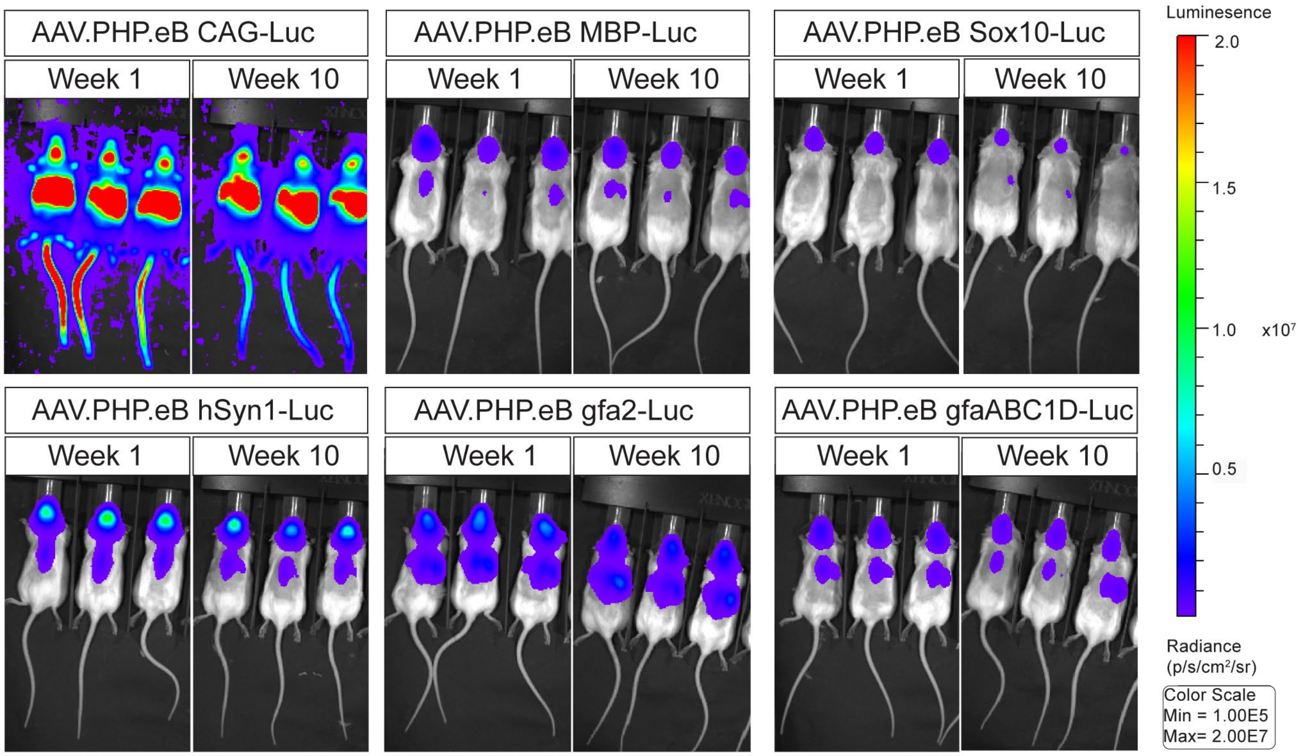

Figure S1. *In vivo* bioluminescence images after one and ten weeks *post*-intravenous AAV administration. Exemplary images of the whole-body bioluminescence in healthy mice after one and ten weeks luciferase expression directed by the general CAG promoter and the CNS directed neural promoters.

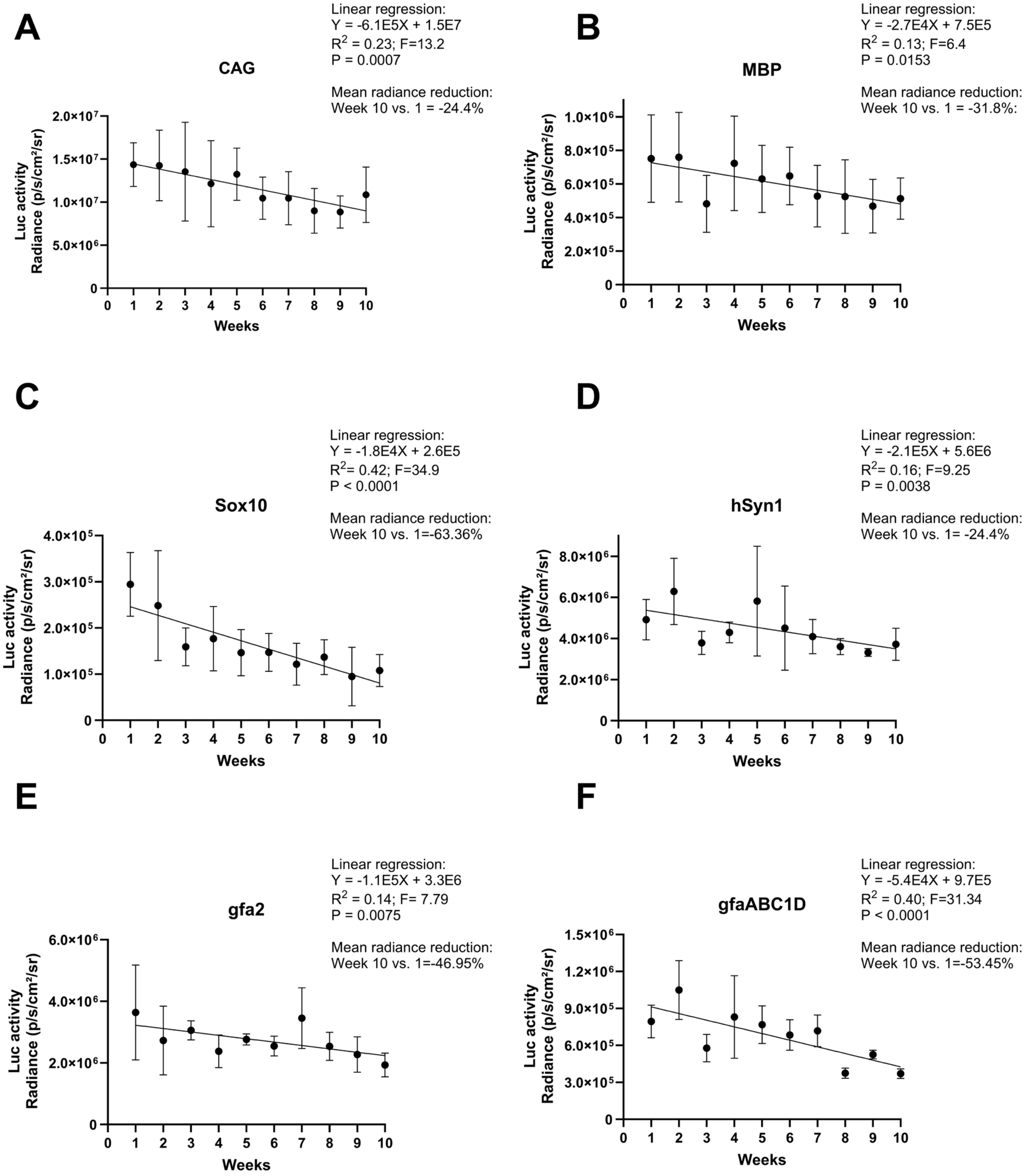

Figure S2. Linear regression analysis of brain-derived bioluminescence measured over the course of ten weeks A-F. Linear regression analysis of all promoters revealed a gradual but significant downwards trend of bioluminescence signal during ten subsequent weekly measurements. All promoters showed a significant decrease in bioluminescence signal at week 10 versus week 1. n=5 per group,57Bl/6N (B6N-Tyrc-Brd/BrdCrCrl), results represent mean (SD).

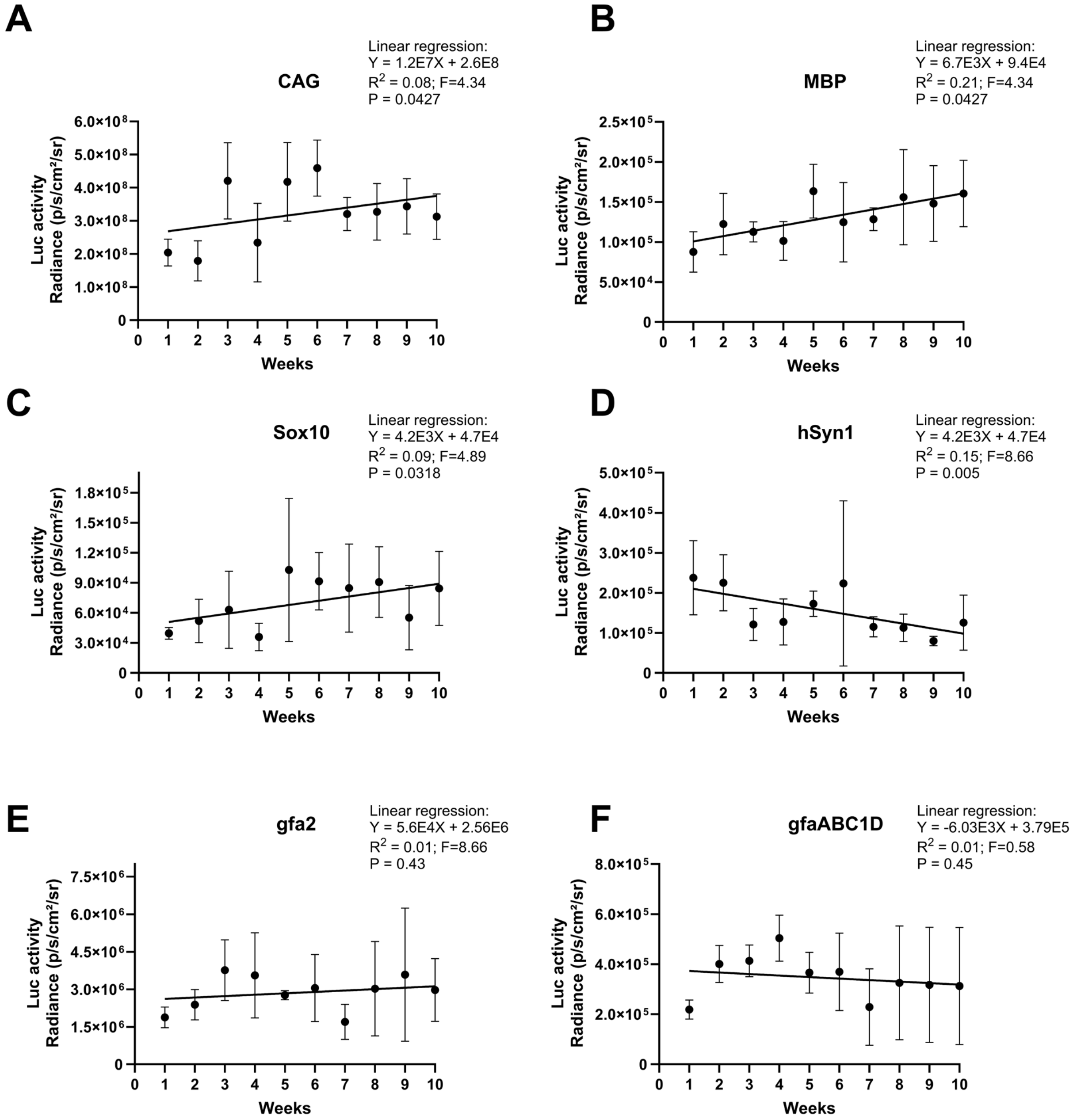

Figure S3. Linear regression analysis of liver-associated bioluminescence measured over the course of ten weeks A-F. Linear regression analysis revealed a gradual but significant upward correlation of the liver-associated bioluminescence signal over the ten weeks measurements period for the CAG, MBP and Sox10 promoters. Liver bioluminescence significantly decreased over the course of the experiment for the hSyn1 promoter. Liver bioluminescence remained stable for gfa2 and gfaABC1D. n=5 per group, C57Bl/6N (B6N-Tyrc-Brd/BrdCrCrl), results represent the mean (SD).

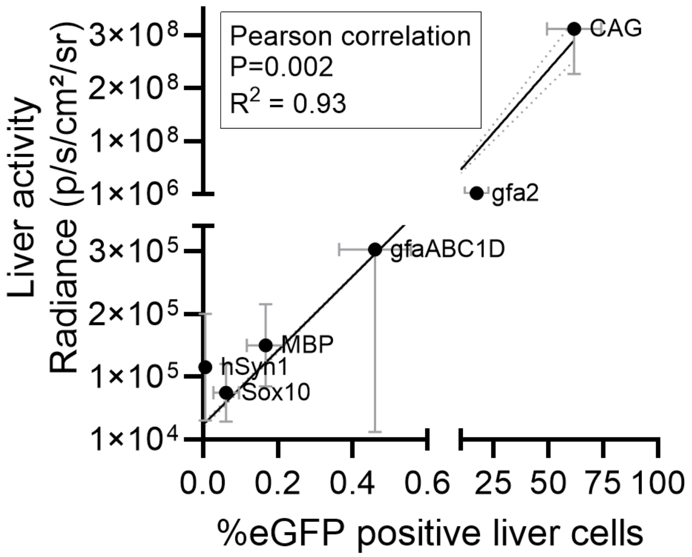

Figure S4. Liver *in vivo* bioluminescence at week ten correlated positively with the proportion of eGFP labeled liver cells. Measured liver bioluminescence was plotted against the percentage of eGFP-positive liver cells. Data represented as mean (SD) in for both axis. Pearson correlation demonstrates a significant correlation (P=0.002; R^2=0.93) between the *in vivo* readout with luciferase and eGFP immunohistochemical analysis of liver expression.

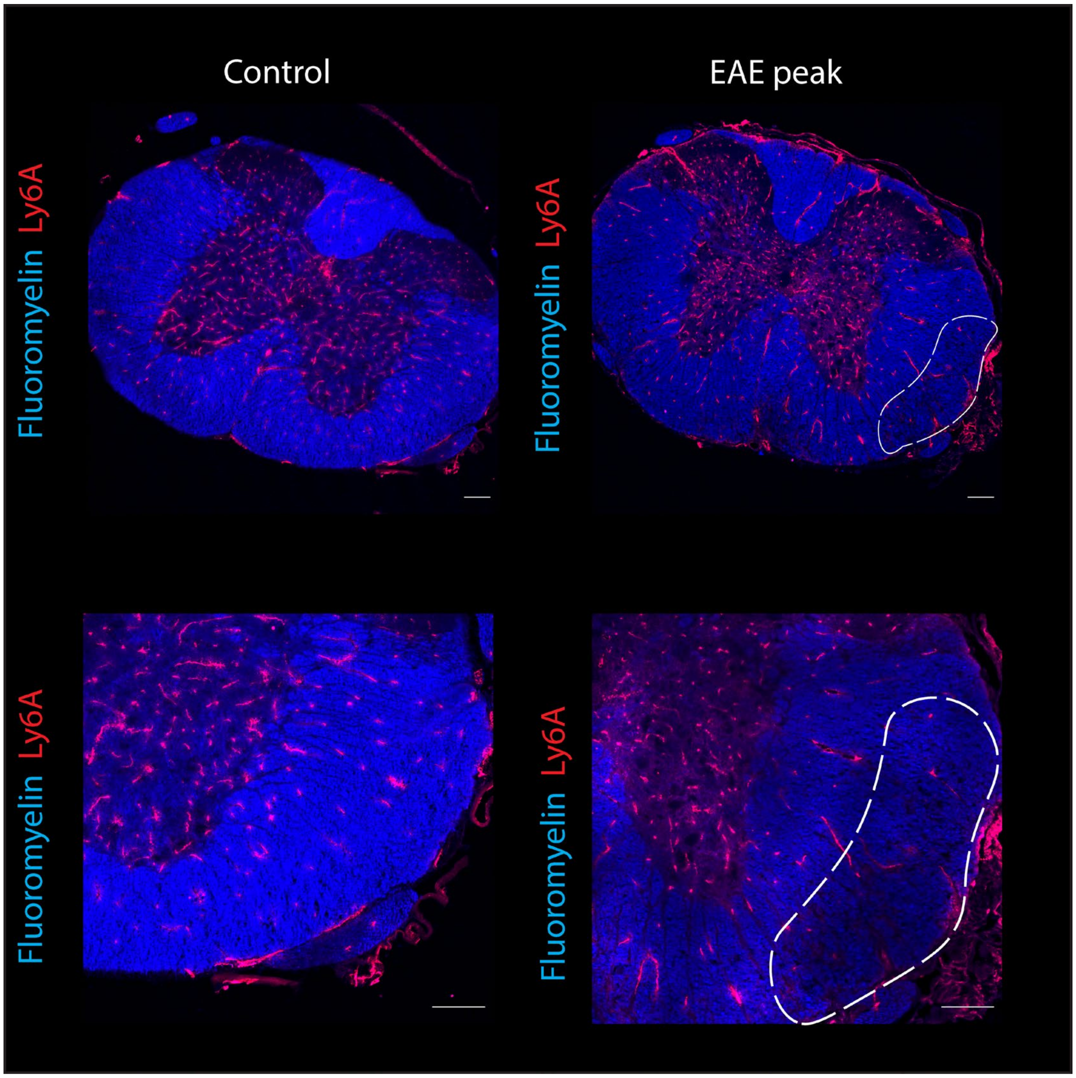

Figure S5. Presence of the endothelial Ly6A receptor in the healthy spinal cord and at the chronic phase of EAE. A. Healthy spinal cord tile scan (Z-stack) showing presence of myelin (fluoromyelin, blue) and the Ly6A receptor (red) in mouse spinal cord grey and white matter tissue. **B**. Higher magnification (x20, dry) of showing presence of myelin (fluoromyelin, blue) and the Ly6A receptor (magenta) in mouse spinal cord grey and white matter tissue. **C**. EAE affected spinal cord at 18 days post $MOG_{35\text{-}55}$ immunization (chronic EAE phase). Stained for fluoromyelin and Ly6A reveal myelin damage and presence of the Ly6A receptor distal, proximal and within the EAE-lesioned spinal cord. **D**. Higher magnification (x20, dry) of the EAE affected spinal cord at 18 days post $MOG_{35\text{-}55}$ immunization (chronic EAE phase) reveals myelin damage and sustained presence of the Ly6A receptor distal, proximal and within the EAE lesioned spinal cord.

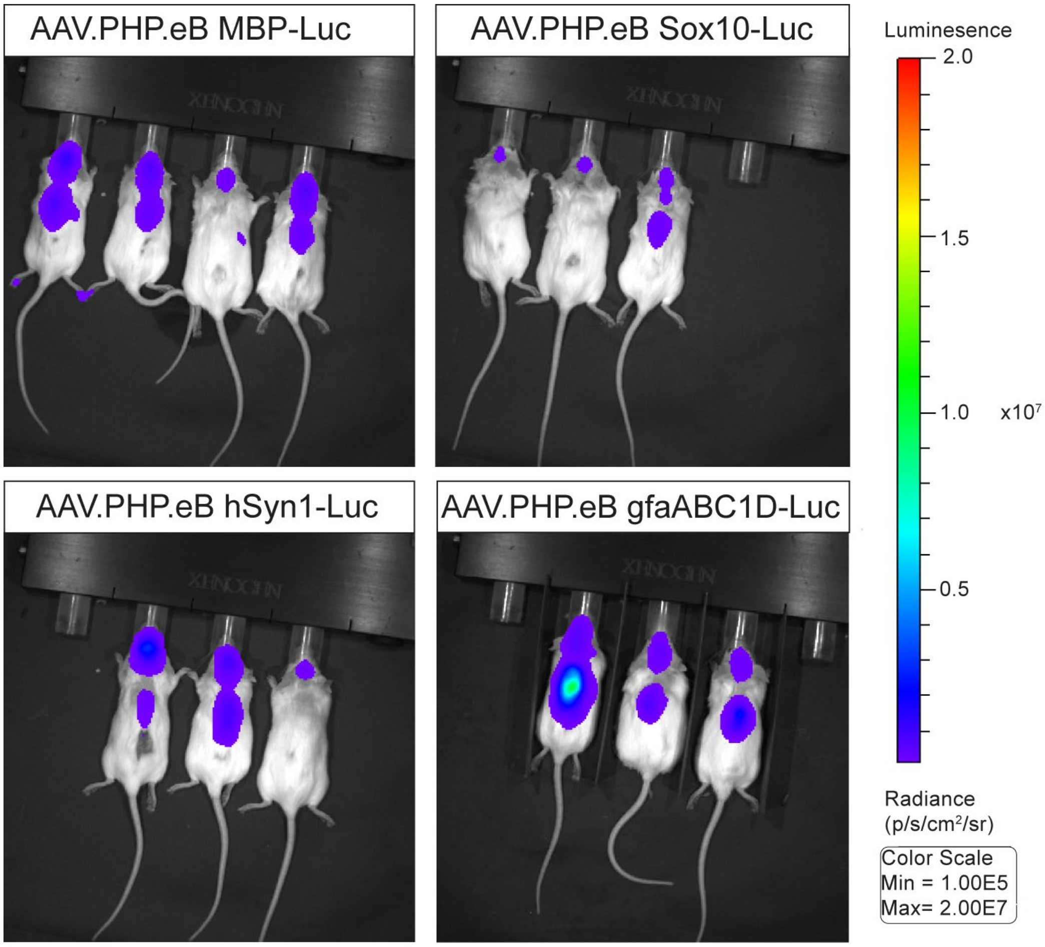

Figure S6. ***In vivo*** **bioluminescence images of EAE mice, four weeks *post*-intravenous AAV administration.** Mice were administered AAV.PHP.eB five days after EAE-induction to monitor the expression of luciferase over the EAE disease course. After four weeks the transgene was still highly expressed during the chronic phase of EAE.

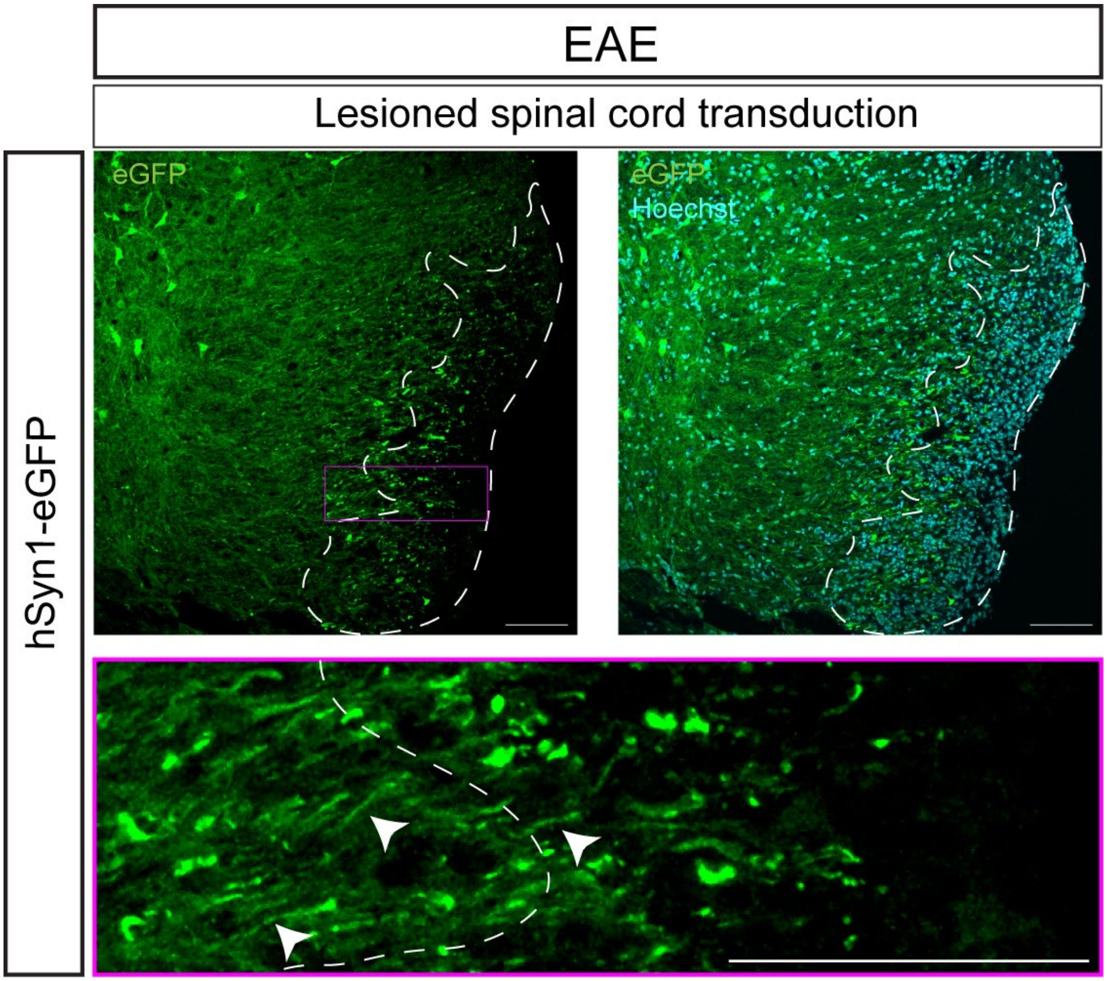

Figure S7. eGFP-labeled axons are present within the EAE lesion but show signs of axonal degeneration at the lesion edge, close to the rim of the tissue. In the spinal cord after four weeks of expression, eGFP-labeled axons can be seen crossing the lesion rim (white arrowheads). Loss and of eGFP-labeled axons near the rim of the tissue suggests axonal degeneration and fragmentation as a result of inflammation. Scale bar = 100 μm.
