# Supplementary material for: Developing a minimally invasive gene therapy for multiple sclerosis
Source: Mol Ther Methods Clin Dev. 2025 Jun 10;33(3):101504. doi: 10.1016/j.omtm.2025.101504 (PMC12536318; doi:10.1016/j.omtm.2025.101504)
Supplement: Document S2. Article plus supplemental information [file mmc2.pdf]

Molecular Therapy
Methods & Clinical Development
Original Article

Developing a minimally invasive gene therapy for multiple sclerosis

Paul J.H. Nijhuis,[1] Maurits Romijn,[1] Roy Honing,[1] Giselle van Zon,[1] Inge Huitinga,[2,3] Fred de Winter,[1] and Joost Verhaagen[1]

[1]Laboratory of Neuroregeneration, The Netherlands Institute for Neuroscience, an Institute of the Royal Netherlands Academy of Arts and Sciences, Meibergdreef 47, 1105 BA Amsterdam, the Netherlands; [2]Laboratory of Neuroimmunology, The Netherlands Institute for Neuroscience, an Institute of the Royal Netherlands Academy of Arts and Sciences, Meibergdreef 47, 1105 BA Amsterdam, the Netherlands; [3]Swammerdam Institute for Life Sciences, University of Amsterdam, Sciencepark 904, 1098 XH Amsterdam, the Netherlands

Multiple sclerosis (MS) is a neurological disease characterized by demyelinating lesions in the CNS. This study investigated whether a minimally invasive adeno-associated virus (AAV) vector (AAV.PHP.eB) can direct transgene expression in CNS cell types relevant to MS, including astrocytes, oligodendrocytes, oligodendrocyte precursor cells (OPCs), microglia, and neurons in experimental autoimmune encephalitis, a widely used MS model. *In vivo* bioluminescence imaging and histological analysis following AAV.PHP.eB-mediated gene delivery in healthy mice using the ubiquitous CAG promoter and five neural promoters (MBP, Sox10, hSyn1, gfa2, and gfaABC1D) revealed long-term, robust, and cell-type-specific activity across the brain and spinal cord. AAV.PHP.eB is capable of traversing the blood-brain barrier in experimental autoimmune encephalitis (EAE) and directs sustained and cell-type-specific transgene expression for the MBP, Sox10, hSyn1, and gfaABC1D promoters. The MBP and Sox10 promoters directed transgene expression in oligodendroglia around and within inflammatory demyelinating lesions, whereas the gfaABC1D promoter directs transgene expression in gray and white matter astrocytes and hSyn1 in neurons. The neural promoters were minimally active in the periphery, with the exception of gfa2. This methodological study is a first step toward the development of minimally invasive gene therapy to promote myelin repair and/or suppress inflammation in MS.

INTRODUCTION

Multiple sclerosis (MS) is a neurological disease characterized by multifocal demyelinating lesions that occur in a spatially and temporally unpredictable manner in the central nervous system (CNS).[1] Gene therapy would provide an innovative and thus far unexplored strategy to stimulate myelin repair through local secretion of therapeutic factors. However, classical gene therapy for the CNS depends on direct intracranial viral vector delivery. When applied to a multifocal disease like MS, this would require multiple and repeated intraparenchymal injections at sites where demyelinating lesions occur. Apart from the fact that it is unrealistic for patients to undergo repeated intraparenchymal viral vector injections, precise targeting of affected sites via direct intracranial administration is very difficult.[2] Given the multifocal, dynamic, and unpredictable nature of MS lesion pathology, effectively dispersing viral vectors throughout the CNS parenchyma, particularly targeting lesions around blood vessels where MS pathology predominantly occurs, would be crucial for therapeutic success.[3] A potential strategy to mitigate invasiveness and increase the chances of therapeutic success involves the use of minimally invasive viral vectors capable of traversing the blood-brain barrier (BBB) in combination with cell-type-specific promoters to direct transgene expression to the cells associated with disease pathology.[4–6] The objective of the experiments described in this article was to develop a gene therapy method that directs cell-type-specific transgene expression in the acute experimental autoimmune encephalomyelitis (EAE) animal model, as a first step to develop a minimally invasive, regenerative gene therapy for MS.

Recombinant adeno-associated viral vectors (AAVs) are the leading choice for gene therapy because of their broad cell tropism and favorable safety profile as demonstrated in human and animal studies.[4,7–10] The BBB, however, prevents efficient systemic delivery of most AAV serotypes to the CNS. Natural AAV serotypes that do cross the BBB, including AAV9 and F-clade derivatives, only show limited entry into the adult CNS.[4,11–14] Site-directed evolution of the AAV9 capsid led to the discovery of AAV.PHP.eB, a capsid with an enhanced capacity to cross the BBB.[6,15] At present, AAV.PHP.eB is the most used vector for systemic delivery to the rodent CNS due to its efficient BBB penetrating capacity, broad CNS cell tropism, and reduced tropism for peripheral organs compared to its parental AAV9 capsid.[5,6,15–17]

Promoters are genomic elements that determine both the level of transgene expression and the cell type in which a transgene is expressed. Systemic administration of AAV.PHP.eB with a strong

Received 11 December 2024; accepted 5 June 2025;
https://doi.org/10.1016/j.omtm.2025.101504.

Correspondence: Paul J.H. Nijhuis, Laboratory of Neuroregeneration, The Netherlands Institute for Neuroscience, an Institute of the Royal Netherlands Academy of Arts and Sciences, Meibergdreef 47, 1105 BA Amsterdam, the Netherlands.
E-mail: p.nijhuis@nin.knaw.nl

© 2025 The Author(s). Published by Elsevier Inc. on behalf of The American Society of Gene and Cell Therapy.
This is an open access article under the CC BY license (http://creativecommons.org/licenses/by/4.0/).

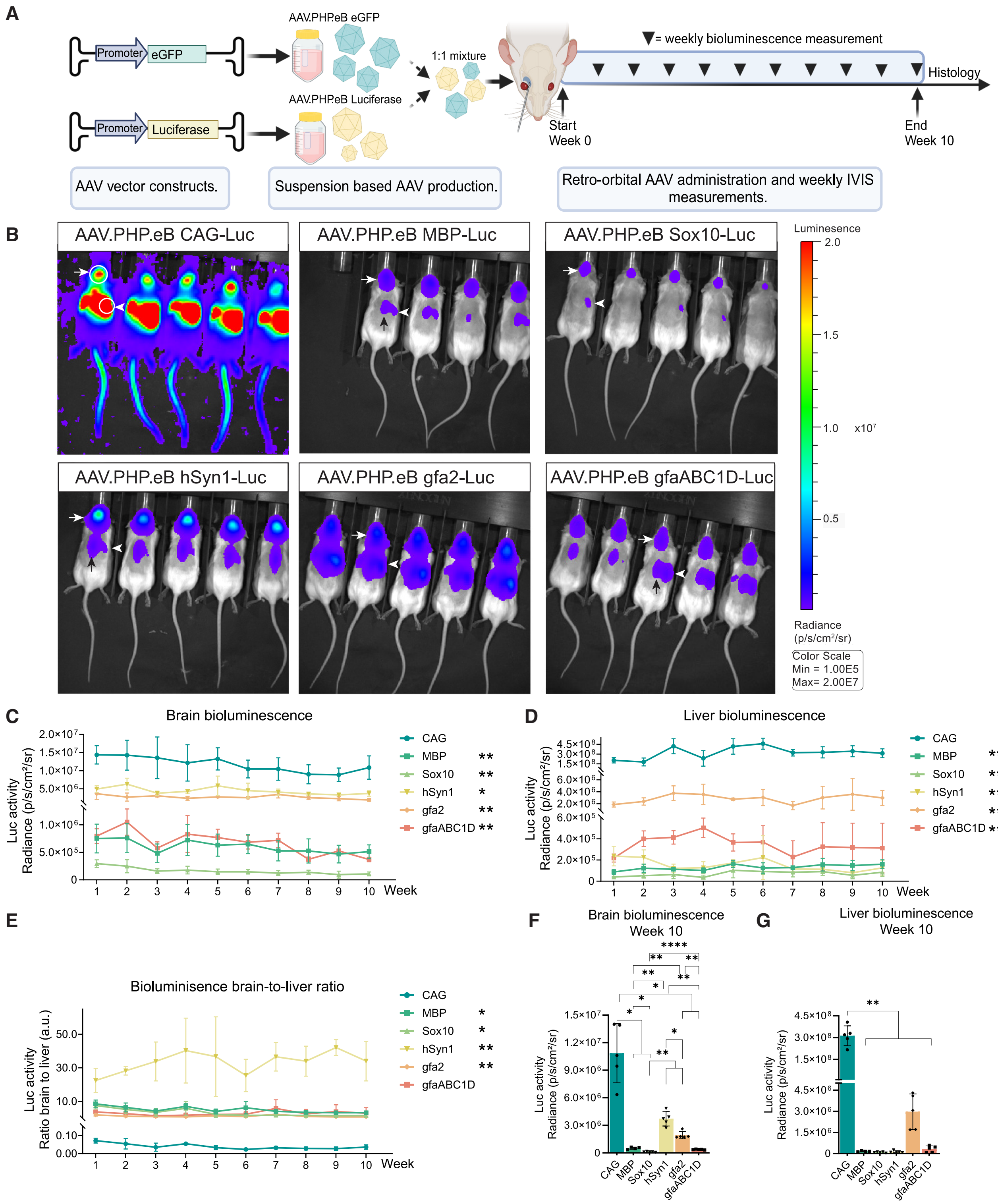

(legend on next page)

ubiquitous promoter, such as CAG (consisting of the CMV early enhancer, chicken β-actin promoter, and β-globin intron), results in widespread transgene expression in the brain and many peripheral organs, including the liver and heart.[5,18] For MS, a disease with multifocal demyelinating lesions throughout the brain, it is essential to target therapeutic gene expression to specific CNS cells associated with the demyelinating lesions, i.e., astrocytes, oligodendrocyte-lineage cells, microglia, and neurons. Moreover, transgene expression in peripheral organs should be minimal to prevent unwanted side effects. Neural promoters that direct gene expression in CNS cells have the potential to enable AAV.PHP.eB-mediated transgene expression in the cells relevant to the disease pathology, while minimizing peripheral transgene expression. The size of the promoter is an important criterium for AAV vector design since AAVs have a maximum packaging capacity of 4.7 kb.[19] Popular compact promoters are the human synapsin 1 promoter (hSyn1), human glial fibrillary acidic protein promoter (gfa2) and its modified version (gfaABC1D), the mouse derived myelin basic protein promoter (MBP), and the mouse SRY-box-containing gene 10 promoter (Sox10). These promoters direct transgene expression to neurons, astrocytes, and oligodendrocytes, respectively.[19–23] To date, there is limited information regarding the spatiotemporal activity and cell-type specificity of these promoters when combined with a systemically administered AAV vector under healthy or neuropathological conditions.

Here, we studied AAV.PHP.eB-mediated gene delivery using these promoters in healthy adult and in EAE mice. The EAE model was chosen as it reliably replicates key pathological features of human MS, including T-cell-mediated microglial activation, focal inflammation, and spinal cord demyelination. The use of AAV.PHP.eB promoter-luciferase and -eGFP reporter constructs enabled longitudinal whole-body *in vivo* bioluminescence imaging combined with a detailed histological analysis of cell-type specificity of the promoters in the CNS of healthy and EAE mice. Taken together, our results show that an AAV.PHP.eB-based minimally invasive gene therapy, in combination with a cell-selective promoter, directs transgene expression to the appropriate neural cell type and significantly reduces transgene expression in peripheral organs. The method developed here is a necessary and important step toward the establishment of an experimental regenerative and/or anti-inflammatory gene therapy for MS.

RESULTS

Whole-body bioluminescence imaging reveals long-term transgene expression following intravenous delivery for all AAV-PHP.eB promoter constructs

Whole-body bioluminescence imaging was used to investigate the long-term spatiotemporal expression profile of the CAG, MBP, Sox10, hSyn1, gfa2, and gfaABC1D promoters. For all promoters a 1:1 mixture of 5×10^{11} AAV-PHP.eB-luciferase and AAV-PHP.eB-eGFP vectors was injected intravenously via the retro-orbital sinus (Figure 1A). The use of a mixture of AAV vectors encoding these two reporter genes allowed longitudinal bioluminescence analysis to investigate whole body distribution of transgene expression and endpoint immunohistochemistry (IHC) to determine the cell types expressing the transgene for each promoter within the same animal. Bioluminescence was measured every week for 10 subsequent weeks. After 10 weeks, the animals were perfusion-fixed, and brain, spinal cord, and peripheral tissues were prepared for IHC. Animals injected with AAV-PHP.eB-CAG vectors showed wide-spread bioluminescence throughout the body. In contrast, animals that received the neural promoter constructs revealed a bioluminescence signal profile largely restricted to the brain (Figure 1B, white arrows) with the exception of the AAV-PHP.eB-gfa2 injected animals, which displayed significant bioluminescence over the abdomen, particularly near the liver area (Figure 1B, white arrowhead). Animals administered with AAV.PHP.eB-hSyn1, -MBP, and -gfaABC1D also displayed bioluminescence signal over the anatomic region of the thoracic spinal cord (Figure 1B, black arrows). For all promoters, the brain-associated bioluminescence signal sustained over the entire observation period of 10 weeks (Figure S1); however, all promoters showed a significant but gradual decline in bioluminescence over time (Figure S2).

The intensity of the bioluminescence signal for each individual promoter is determined by a number of parameters, including (1) differential cellular tropism of AAV.PHP.eB for various neural cell types, (2) the number of transduced cells expressing the transgene, and (3) the level of transcriptional activity of the promoter in individual cells.

Figure 1. Whole-body bioluminescence imaging shows long-term reporter expression and preferential transgene expression in the CNS for neural promoters compared to a ubiquitous CAG promoter

(A) Schematic overview of the study design. Luciferase and eGFP reporter constructs were individually packaged in AAV.PHP.eB and co-delivered as a 1:1 mixture of 5×10^{11} gc/mouse per vector via the retro-orbital sinus. Luciferase activity was measured weekly for 10 weeks. $N = 5$ animals per group. (B) Representative bioluminescence images for each promoter group at 10 weeks post-AAV.PHP.eB administration demonstrated widespread expression throughout the body for CAG-luciferase-injected animals. In contrast, the MBP, Sox10, hSyn1, and gfaABC1D groups exhibited more brain-restricted expression (arrows) with less liver activity (arrowheads). Example regions of interest (ROIs) are shown for the first mouse in the CAG group (white circles). The thoracic spinal cord signal was visible in the MBP, hSyn1, and gfaABC1D groups (black arrows). (C) The brain-associated bioluminescence signal revealed sustained expression for all neural promoters for at least 10 weeks. Over the course of 10 weeks, all neural promoters had significantly lower brain-derived bioluminescence compared to the general CAG promoter. Data are presented as mean (SD). Statistics: Brown-Forsythe and Welch ANOVA tests with Dunnett's T3 multiple comparison correction. (D) Liver-associated bioluminescence revealed that CAG promoter activity was significantly higher than that of neural promoters over the course of 10 weeks ($p < 0.001$). Data are presented as mean (SD). Statistics: Brown-Forsythe and Welch ANOVA tests with Dunnett's T3 multiple comparison. (E) Except for gfaABC1D, the brain-to-liver bioluminescence ratio measured over 10 weeks revealed significantly higher brain-specific activity for the neural promoters compared to the low ratio (<0.1) observed for the CAG promoter. Data are presented as mean (SD). Statistics: Brown-Forsythe and Welch ANOVA tests with Dunnett's T3 multiple comparison correction. (F) Except for hSyn1, after 10 weeks of expression, brain-derived bioluminescence was significantly higher for the CAG promoter compared to the neural promoters. (G) Liver activity at 10 weeks remained significantly higher for the CAG promoter compared to the neural promoters ($p < 0.01$).

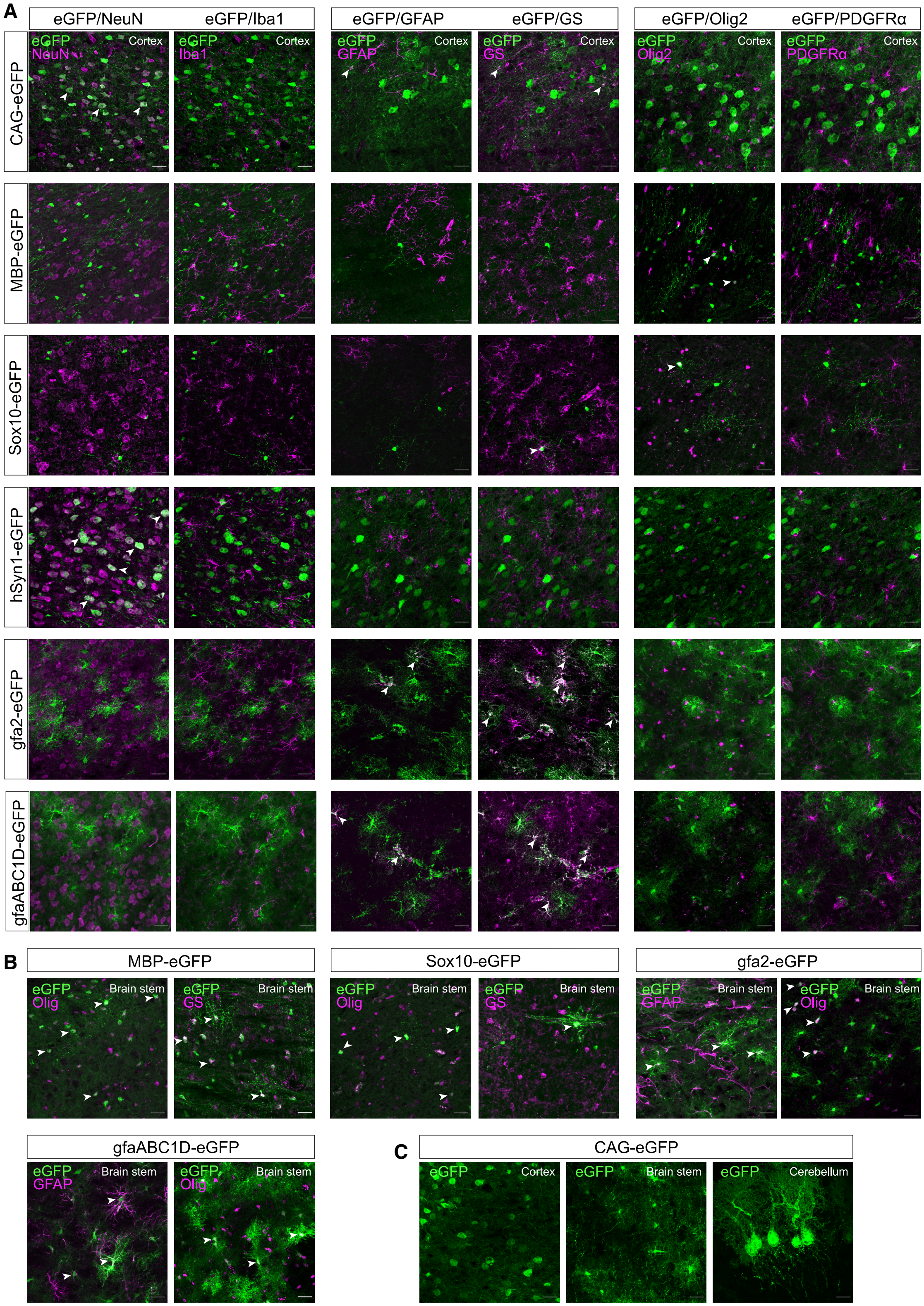

(legend on next page)

Moreover, the bioluminescence signal is partially scattered and absorbed by overlaying tissue, which results in diminished signal from transduced cells deeper in the tissue.[24] Over the course of 10 weeks, the CAG promoter showed the highest bioluminescence signal in the brain and in the abdomen compared to the neural promoters (Figures 1C and 1F). This is in line with the expected ubiquitous activity of the CAG promoter not only in different cell types including neurons and astrocytes but also in non-neural peripheral cells like hepatocytes. For the neural promoters, the highest levels of brain luciferase activity were observed in the hSyn1 and gfa2 groups followed by MBP and gfaABC1D. The Sox10 group showed the lowest luciferase activity. After 10 weeks of expression, bioluminescence imaging of the two astrocyte promoters, gfa2 and gfaABC1D, revealed a significant 5-fold difference in bioluminescence signal between these promoters ($p < 0.01$; Figure 1F). This observation implies either a reduced number of astrocytes expressing the luciferase transgene in the gfaABC1D group or a lower promoter activity compared to gfa2. Taken together, the neural promoters remained active *in vivo* for at least 10 weeks and demonstrated lower brain luciferase activity compared to the ubiquitous CAG promoter.

Systemic administration of AAV.PHP.eB also results in transduction outside the CNS. Therefore, the peripheral bioluminescence activity, in particular in the liver area, was analyzed. This is especially relevant in the context of a therapeutic transgene, since peripheral overexpression could result in unwanted side effects. To exclude potential signal from luciferase expression in the thoracic spinal cord, a region of interest was placed over the liver's right superior lobe to measure the liver-associated bioluminescence (Figure 1B). Over a period of 10 weeks, liver-associated luciferase activity for the CAG group was significantly higher compared to MBP (2,540-fold), Sox10 (4,600-fold), hSyn1 (2,090-fold), gfa2 (112-fold), and gfaABC1D (931-fold) (relative to CAG: $p < 0.001$; Figures 1D and 1G). Liver bioluminescence gradually increased during the 10-week observation period for the CAG group and the two oligodendroglial promoter groups, MBP and Sox10. Interestingly the hSyn1 group showed a nearly 50% decline of liver-associated bioluminescence over the course of 10 weeks. The liver bioluminescence remained stable over the 10-week period for the gfa2 and gfaABC1D groups (Figure S3).

The brain-to-liver signal ratio further shows the acquired preferential transgene expression in the brain with the neural promoters compared to the general CAG promoter (Figure 1E). Over the course of 10 weeks, the neural promoters, except for gfaABC1D, had a significantly higher brain-to-liver ratio compared to the CAG promoter. For CAG only, the liver bioluminescence was consistently higher compared to the brain-derived signal with a signal ratio below 0.1. Overall, whole-body *in vivo* bioluminescence measurements revealed that AAV-PHP.eB directed robust transgene expression in the brain for 10 weeks, with a gradual yet significant decrease in bioluminescence signal over time.

Cellular transgene expression profile following AAV.PHP.eB-promoter-mediated gene delivery

Sagittal sections of the cortex, corpus callosum, brain stem, cerebellum, and transverse sections of the spinal cord were used to visualize and quantify the cell types that expressed the reporter transgene eGFP following transduction with the AAV.PHP.eB promoter constructs. Immunohistochemical markers targeting specific cell types were used to study co-localization with eGFP (Figure 2A). NeuN, a pan-neuronal marker, stains nearly all neurons. Glial fibrillary acidic protein (GFAP) labels a large sub-population of astrocytes, though not all. Therefore, a glutamine synthetase (GS) marker was included to capture a more complete profile of transduced astrocytes. Olig2 marks oligodendroglia, PDGFRα identifies oligodendrocyte precursor cells (OPCs), and Iba1 marks microglia. The selected markers enabled detailed assessment of the cell type specificity of both CAG and the neural promoters. Cellular specificity for the neural promoters was defined as the proportion of eGFP-labeled cells that were also positive for a particular cell-type-specific marker.

Ubiquitous CAG promoter activity varies strongly between neurons and astrocytes depending on nervous system area

In the CAG group, transgene expression was predominantly observed in neurons and astrocytes throughout the brain and spinal cord (Figures 3A–3G). Interestingly, the activity of the CAG promoter in neurons and astrocytes was different between the five brain regions analyzed. In the cortex, transgene expression showed predominant co-localization with NeuN (90.3% ± 3.7%; Figures 2A and 3A) but hardly any overlap with GFAP (0.8% ± 0.7%), GS (2.3% ± 2.0%), and the combined markers GFAP/GS (0.6% ± 0.6%; Figures 3B–3D). In contrast, in the brain stem only 9.5% ± 4.7% eGFP-labeled cells co-localized with NeuN, whereas 42.3% ± 14.9% of eGFP-expressing cells co-localized with the astrocyte marker GFAP and

Figure 2. AAV-PHP.eB-mediated delivery of neural promoter constructs directs cell-type-specific transgene expression in the brain

(A) Immunohistochemistry for eGFP (green) and cell-type-specific markers (magenta) on sagittal brain sections for each promoter group 10 weeks post-AAV application. Microscope settings for intensity are not equal between experimental groups to improve visualization of transduced cells and co-localizations. In the cortex, the ubiquitous CAG promoter directed eGFP expression predominantly in neurons (NeuN) and in a small proportion of astrocytes (GFAP and GS). The MBP and Sox10 promoters were active in oligodendrocytes based on the cellular morphology of the transduced cells and Olig2 co-localization. Astrocyte-specific eGFP expression by the gfa2 and gfaABC1D promoters was evident through strong co-localization with the astrocyte markers GFAP and GS. None of the promoters were active in Iba1-positive microglia. Arrows point to double-labeled cells (white). (B) In the brain stem, cellular morphology revealed non-cell-type-specific co-localization of the astrocytic marker GS and oligodendrocyte marker Olig2. For both the MBP and Sox10 groups, morphologically defined oligodendrocytes co-localized with Olig2 as expected (left image). However, there was also co-localization of oligodendrocytes with GS-positive cells (right image). Likewise, in the gfa2-and gfaABC1D-groups, eGFP-expressing astrocytes that co-localized with GFAP (left image), frequently co-localized with the Olig2 marker (right image), although the numbers of Olig2-positive astrocytes varied between nervous system areas. (C) The CAG promoter demonstrated high-neuron- and low-astrocyte-specific expression in the cortex, but high-astrocyte- and low-neuron-specific activity in the brain stem. Additionally, Purkinje cells were positively labeled with the CAG promoter in the cerebellar gray matter. Scale bars, 25 μm.

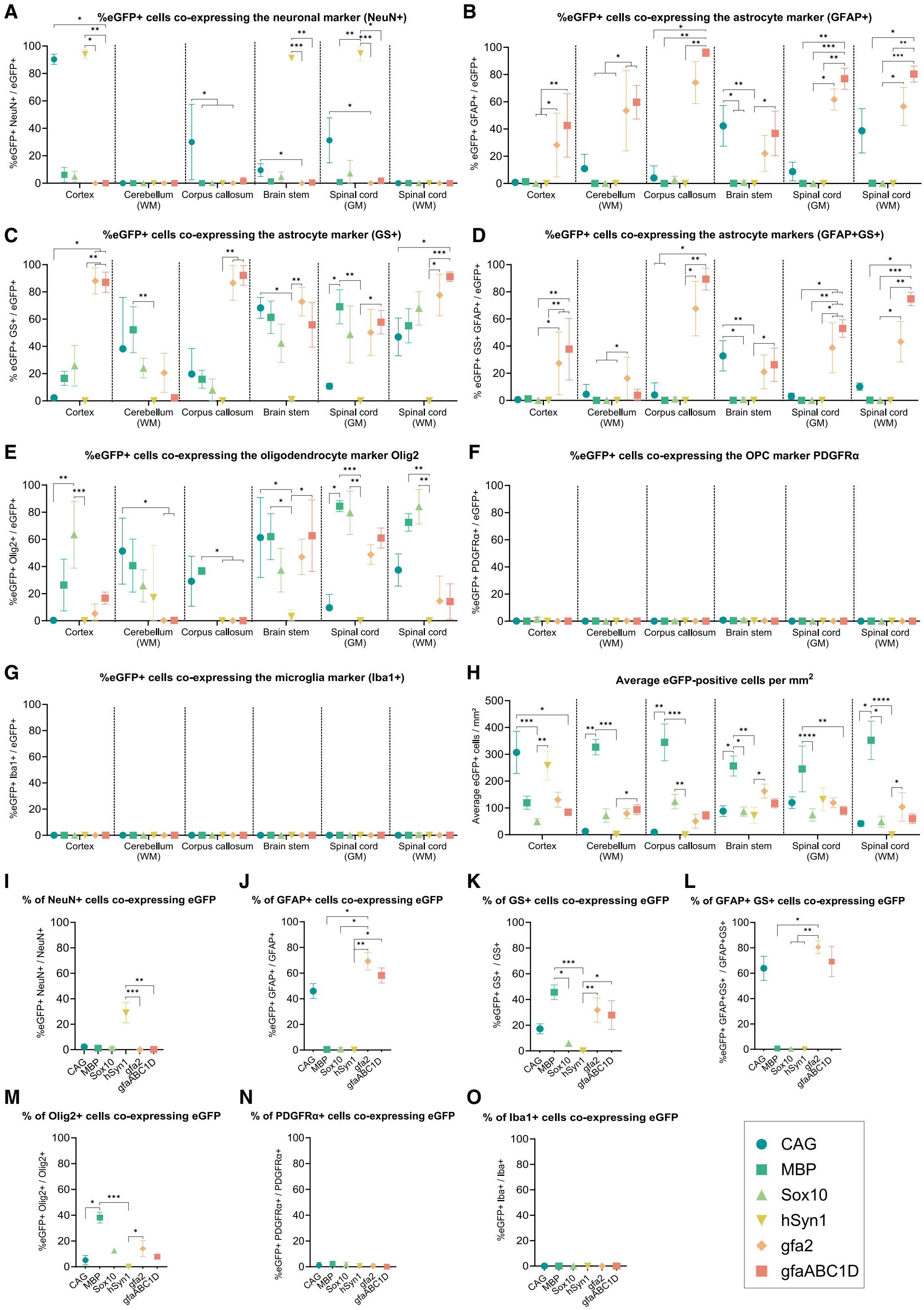

(legend on next page)

68.3% ± 7.6% with GS. Proportion wise, only 2.2% ± 1.4% of all brain stem neurons expressed eGFP, whereas a large proportion of astrocytes expressed eGFP based on co-localization with the two astrocyte markers (GFAP: 46.0% ± 5.8%; GS: 17.1% ± 3.9%) and both markers combined (GFAP/GS; 63.9% ± 9.5%). In the dorsal horn, 31.3% ± 16.4% of eGFP-expressing cells turned out to be neurons and 8.7% ± 6.8% of the transduced cells co-localized with GFAP-positive astrocytes. In the spinal cord white matter, 46.9% ± 13.8% of the transduced cells also expressed GS, 38.7% ± 16.3% co-localized with GFAP-positive cells, and 37.4% ± 11.9% co-localized with Olig2-expressing cells. Importantly, we observed poorer overlap of the astrocyte markers GS and GFAP in the cerebellar white matter, brain stem, and spinal cord and increased staining of ovoid cells. This observation is in line with literature showing that in certain CNS regions, oligodendrocytes increase GS expression dependent on the CNS area.[25–28] Therefore, co-localization with the GFAP marker provides a more reliable estimate of the proportion of eGFP-expressing astrocytes in the brain stem, even though this may reflect only a subset of the astrocyte population (Figure 3J). Both the spinal cord white matter and cerebellar white matter contained eGFP-positive astrocytes, whereas in line with the absence of neurons in the white matter, we did not find co-labeling of eGFP and NeuN in these areas of the spinal cord and cerebellum. In the cerebellar gray matter, the CAG promoter resulted in eGFP labeling of Purkinje cells and neurons of the deep cerebellar nuclei (Figure 2C). Surprisingly, in a few animals we did observe eGFP-expressing cells co-colocalized with a few neuronal somata in the corpus callosum. Although rare, the presence of neuronal cell bodies in the corpus callosum has been reported before.[29]

Co-labeling with Olig2 revealed that 29.1% ± 18.4% of the transduced cells in the corpus callosum were oligodendrocytes. In the brain stem, 61.3% ± 29.4% of the eGFP-positive cells co-localized with Olig2. In the cortex, there was minimal eGFP/Olig2 co-localization (Figure 3E). It is important to mention that the morphology of the eGFP-expressing cells in the brain stem virtually all resembled astrocytes, not oligodendrocytes (Figure 2C). This observation is likely explained by the fact that approximately 75% of S100β+ astrocytes in the medulla oblongata and 90% in the spinal cord also express Olig2, whereas in the cortex this proportion is only around 10%.[30]

No overlap of CAG-eGFP and microglia marker Iba1 was found in any of the analyzed nervous system areas (Figures 3G and 3O). Taken together, the CAG promoter drives transgene expression in many neurons and astrocytes, and transgene expression is minimal in oligodendrocytes. Striking region-specific differences were observed in the cellular expression profile of this promoter, with a preference for neurons in the cortex and astrocytes in the brain stem.

The MBP and Sox10 promoters are predominantly oligodendroglia-specific but show faint transgene expression in a small number of neurons and astrocytes

In all examined CNS regions, AAV.PHP.eB-mediated transgene expression with the MBP and Sox10 promoter revealed high specificity of both promoters for cells of the oligodendrocyte lineage. In the cortical gray matter, eGFP-labeled cells predominantly co-expressed Olig2, with 26.3% ± 19.1% in the MBP group and 63.4% ± 24.6% in the Sox10 group (Figure 3E). In the spinal cord white matter, 72.5% ± 6.5% of transduced cells co-localized with the Olig2 marker for MBP and 84.1% ± 12.7% for Sox10. In the MBP group, a large proportion of eGFP+ cells in the corpus callosum were also Olig2 positive (36.7% ± 2.6%). Additionally, in the Sox10 group, we observed many eGFP-expressing cells in the corpus callosum. Unfortunately, due to inconsistent Olig2 staining in the corpus callosum of animals in this group, only a minor subset of samples could be reliably double-labeled, which led to an unreliable quantification of eGFP/Olig2 co-labeling (data not shown). However, based on morphological features and the absence of co-localization with other markers, these cells were not astrocytes, microglia, or neurons (Figures 3A–3G). Although the proportions of eGFP/Olig2-expressing cells for both groups were rather low in certain CNS areas, virtually all eGFP-expressing cells morphologically resembled oligodendrocytes rather than other glial cell types (Figures 2A and 2B). This observation can be explained by two factors: (1) Olig2 expression decreases as oligodendrocytes mature,[31,32] making it difficult to detect mature Olig2-positive oligodendrocytes expressing eGFP, and (2) detecting Olig2 via IHC is technically challenging as variations in tissue fixation or freezing can significantly affect Olig2 detection, especially in the absence of antigen retrieval, which is currently not feasible when eGFP co-localization is required. Thus, variations in eGFP/Olig2 co-localization between CNS regions do not reflect a spatial change in oligodendrocyte specificity for the MBP and Sox10 promoters.

Activity of the MBP and Sox10 promoters in OPCs was determined by counting the number of transduced cells that co-localized with the OPC marker PDGFRα. The Sox10 promoter directed eGFP expression in a small proportion of OPCs in the cortex (1.4% ± 1.4%; Figure 3F). This low percentage of transduction could be due to either low promoter activity in OPCs or low tropism of AAV.PHP.eB for OPCs.

Neuronal activity of the MBP and Sox10 promoters was quantified by counting the number of eGFP-positive cells that co-localized with NeuN. In the cortex, a small fraction of eGFP-positive cells turned out to be neurons in the MBP group (6.1% ± 5.4%) and 4.8% ± 3.9% in the Sox10 group. Also in the brain stem and spinal cord, transduced neurons were detected in the Sox10 group

Figure 3. Quantitative analysis shows predominant cell-specific transgene expression for all neural promoters

(A–G) Graphs show for each promoter the proportion of eGFP-positive cells that co-localized with NeuN, GFAP, GS, GFAP/GS, PDGFRα, Olig2, and Iba1-expressing cells in the indicated CNS areas. Data represented as mean (SD). *N* = 3–8 animals per group. Statistics: Kruskal-Wallis test with Dunn's correction for multiple comparisons. (H) Average number of eGFP-expressing cells/mm^2 in the indicated CNS areas. Data represented as mean (SD). *N* = 3–8 animals per group. Statistics: Kruskal-Wallis test with Dunn's correction for multiple comparisons. (I–O) Proportion of transduced neurons and glia in the brain stem based on co-localization with the cell-type markers. Data are presented as mean (SD). *N* = 3–5 animals per group. Statistics: Kruskal-Wallis test with Dunn's correction for multiple comparisons.

(Figure 3A). These observations are in line with previous studies that demonstrated weak activity of both oligodendrocyte promoters in neurons.[21,33,34]

The activity of MBP and Sox10 in astrocytes was determined by quantifying the number of eGFP-expressing cells that co-localized with the astrocyte markers GFAP and GS. In the cortex, a small fraction of the transduced cells was revealed to be GFAP-positive astrocytes for the MBP promoter (1.3% ± 1.0%; Figure 3B). For both oligodendroglial promoters, there was a relatively large amount of co-localization between eGFP-expressing cells and the GS marker in all analyzed CNS areas (Figure 3C). However, virtually all eGFP-expressing cells that co-localized with the GS marker exhibited an oligodendrocyte-like morphology and did not co-label with the GFAP marker (Figures 2A, 2B, and 3B–3D). As mentioned previously and consistent with the literature, in addition to astrocytes, the GS marker labeled oligodendrocytes in the cerebellar white matter, brain stem, and spinal cord.[25–28] Nonetheless, the absence of co-localization with the astrocyte marker GFAP in these regions further supports that these eGFP/GS co-labeled cells were predominantly oligodendrocyte lineage cells and not astrocytes.

In the MBP promoter group, higher numbers of transduced cells/mm^2 were quantified in most CNS regions compared to the Sox10 group. For example, the number of positive cells per area in the brain stem and spinal cord white matter were significantly 3-fold and 7-fold higher in the MBP group compared to the Sox10 group, respectively ($p < 0.05$; Figure 3H).

Expression of eGFP in microglia cells was analyzed by counting the number of positive cells that localized with the microglial marker Iba1. None of the eGFP-positive cells in the MBP and Sox10 groups were microglia based on the absence of co-localization with Iba1.

Taken together, our data revealed the MBP and Sox10 promoters to be predominantly active in oligodendroglia, although a relatively small degree of transgene expression in neurons and astrocytes was detected with both promoters.

The hSyn1 promoter is highly neuron-specific throughout the adult mouse CNS

Neurons were highly selectively targeted in the AAV-PHP.eB hSyn1 group. In the cortex, brain stem, and spinal cord, more than 90% of the eGFP-positive cells expressed NeuN (cortex: 94.2% ± 3.4%; brain stem: 91.3% ± 1.1%; spinal cord: 94.7% ± 5.1%; Figure 2A and 3A). Notably, all eGFP-expressing cells morphologically resembled neurons. Since not all neurons express NeuN equally, co-localization proportions are likely slight underestimations and do not reflect transgene expression in non-neuronal cells.[35]

In the brain stem, 29.1% ± 8.1% of all NeuN-positive neurons co-expressed eGFP (Figure 3I). As expected, hSyn1 resulted in strong eGFP labeling of axons in white matter. In the cerebellum, the hSyn1 promoter selectively drove eGFP expression in deep cerebellar nuclei neurons and interneurons in the molecular cellular layer (data not shown). Although rare, eGFP expression was detected in Olig2-positive cells in the cerebellar white matter and brain stem (Figure 3E). Taken together, these data demonstrate that the hSyn1 promoter is neuron-specific throughout the CNS in the context of a systemically administered AAV vector.

The gfa2 and gfaABC1D promoters showed high astrocyte specificity and robust transgene expression across all analyzed CNS regions

Histological co-localization for the gfa2-EGFP and gfaABC1D-EGFP groups revealed that both promoters directed astrocyte-specific transgene expression following systemic delivery (Figure 2A). As mentioned previously, the GFAP marker labels only a sub-population of homeostatic astrocytes. Therefore, glutamine synthetase (GS) was included as a second astrocyte marker. Astrocyte specificity of the gfa2 and gfaABC1D promoters was determined by quantifying the degree of co-localization of eGFP-expressing cells with the GFAP, GS, and both markers combined.

Overlap of eGFP-expressing cells using the two astrocyte markers GFAP and GS demonstrated no significant difference in the astrocyte specificity of both promoters in all CNS regions (Figures 3B, 3C, and 3D). Differences in the co-localization of eGFP-labeled cells between the two astrocyte markers are mostly caused by the broader expression of GS, which is present in a larger astrocyte population compared to GFAP. For example, in the cortex there was a large degree of overlap with the GS marker (gfa2: 88.1% ± 9.6%; gfaABC1D: 87.0% ± 7.5%), and fewer co-localizations were observed with the GFAP marker (gfa2: 28.2% ± 23.5%; gfaABC1D: 42.6% ± 23.3%). In the brain stem, a large proportion of astrocytes expressed eGFP based on the GFAP marker (gfa2: 69.3% ± 6.9%; gfaABC1D: 58.2% ± 5.9%; Figure 3J), GS (gfa2: 31.7% ± 9.4%; gfaABC1D: 27.8% ± 11.2%; Figure 3K), or a combination of the astrocyte markers (gfa2: 80.5% ± 5.0%; gfaABC1D: 69.1% ± 11.8%; Figure 3L). Notably, even though we included two astrocyte markers, there were still eGFP-expressing cells with astrocyte morphology that did not overlap with either of the markers, resulting in a slight underestimation of the proportion of transduced astrocytes. These data demonstrate both astrocyte promoters to be highly active in astrocytes.

Next, the activity of the gfa2 and gfaABC1D promoters in oligodendroglial cells was quantified by counting the number of transduced cells that co-expressed Olig2. A relatively large proportion of the eGFP-positive cells co-localized with Olig2-positive cells in the brain stem (gfa2: 47.0% ± 13.0%; gfaABC1D: 62.7% ± 26.4%) and spinal cord gray (gfa2: 48.8% ± 7.3%); gfaABC1D: 61.0% ± 7.4%) and white matter (gfa2: 14.6% ± 18.4%; gfaABC1D: 14.2% ± 13.0%). Importantly, for both groups the eGFP/Olig2-expressing cells morphologically resembled astrocytes but not oligodendrocytes (Figure 2B). Others have similarly reported strong Olig2 expression in region-specific subpopulations of astrocytes.[30] Finally, both astrocyte promoters were inactive in OPCs, microglia, and virtually inactive in

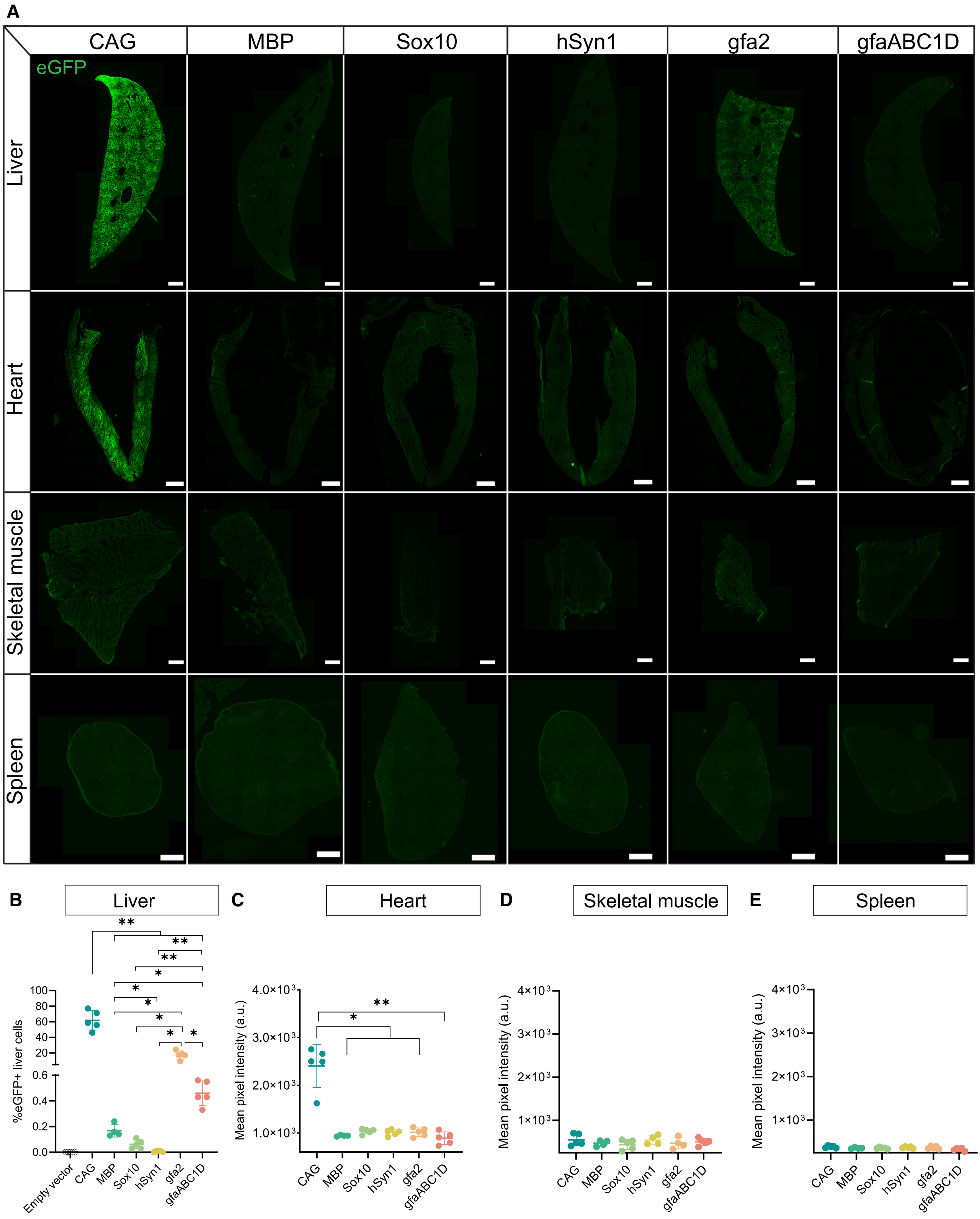

(legend on next page)

neurons (Figures 3A, 3F, and 3G). Taken together, these data demonstrate that both gfa2 and gfaABC1D drive astrocyte-specific transgene expression in all analyzed CNS areas when delivered systemically via AAV.PHP.eB to the adult mouse CNS.

Neural promoters show minimal peripheral activity compared to the ubiquitous CAG promoter after systemic AAV.PHP.eB delivery

Next, we quantified to what extent CAG and the neural promoters were active in the liver, heart, skeletal muscle, and spleen (Figure 4A). In line with the bioluminescence data, the CAG promoter was most active in the liver, with 61.7% ± 12.4% of the liver cells expressing eGFP (Figure 4B). As expected from the bioluminescence data, histology confirmed that all neural promoters were significantly less active in liver cells compared to the ubiquitous CAG promoter ($p < 0.01$; Figure 4B). No fluorescent liver cells were detected in healthy control animals that received empty AAV.PHP.eB. Among the neural promoters, hSyn1 was least active in the liver, with only 0.006% ± 0.005% of liver cells expressing eGFP. The MBP promoter labeled 0.17% ± 0.05% of liver cells, and the Sox10 labeled 0.06% ± 0.03% of the liver cells. The gfaABC1D promoter directed eGFP expression in only 0.46% ± 0.096% of the liver cells, whereas the gfa2 promoter resulted in eGFP expression within a significantly higher fraction of livers cells (17.3% ± 5.4%; $p < 0.05$; Figure 4B). Liver bioluminescence at the time of sacrifice correlated positively with the proportion of eGFP-expressing liver cells (Figure S4). The CAG promoter was highly active in the heart (Figure 4C). None of the neural promoters resulted in detectable eGFP fluorescence in cardiac muscle, skeletal muscle, and spleen (Figure 4A and 4C–4E).

Systemically administered AAV.PHP.eB in EAE sustains CAG and neural promoter activity in the CNS

After showing long-term *in vivo* transgene expression directed by the neural promoters in healthy mice (i.e., wild-type mice), we examined whether the promoters remained active and cell-type-specific in the EAE mouse model for MS. The gfa2 promoter was excluded from further testing in the EAE mouse model and deemed unsuitable for future systemic nervous system gene therapy studies since this promoter was highly active in the liver.

Post-intravenous administration, AAV.PHP.eB traverses the BBB through binding of the endothelial lymphocyte antigen 6 complex locus A (Ly6A) receptor.[36–38] Given the damage to the BBB in EAE[39] and the absence of data in the literature on the sustained presence of Ly6A in this context, we first confirmed the presence of the Ly6A receptor in the endothelial cells of EAE mice (Figure S5).

AAV.PHP.eB luciferase and eGFP vectors were administered in a 1:1 mixture of 5 x 10^{11} gc/per vector via the retro-orbital sinus. AAV was administered 5 days after EAE induction to enable whole-body *in vivo* monitoring of promoter activity throughout the disease progression, from peak EAE to the chronic phase (Figure 5A).

Both CAG and the neural promoters remained active and directed robust transgene expression for 4 weeks in the brains of EAE-affected mice (Figures 5B–5F and S6). Interestingly, all promoters exhibited a brain-associated peak in bioluminescence signal that coincided with the apex of EAE neurological scores. Importantly, the peak in bioluminescence during the early phase of EAE likely reflects increased BBB permeability, leading to higher luciferin levels entering CNS, rather than an increase in promoter activity caused by EAE pathology.[39,40] The Sox10, hSyn1, and gfaABC1D promoters revealed similar bioluminescence profiles, characterized by a bioluminescence peak during the first week, followed by a decline and subsequent stabilization of the bioluminescence signal over the next 3 weeks. Increased bioluminescence in the first week was 4-, 7-, and 8-fold higher compared to the signal in the second week for Sox10, hSyn1, and gfaABC1D, respectively (Figures 5D–5F). After the bioluminescence signal peak in the first week, the bioluminescence profiles in the chronic EAE phase resembled the signals previously measured in healthy mice. The MBP promoter revealed a significant bioluminescence peak in the first 2 weeks after which the bioluminescence signal stabilized to levels observed in healthy mice in the subsequent weeks in the chronic phase of EAE ($p < 0.05$; Figure 5C). Overall, AAV.PHP.eB efficiently transduces the CNS in the EAE mouse model for MS, with all promoters directing transgene expression for at least 4 weeks.

Neural promoter specificity in the spinal cord of EAE mice

Next, we investigated whether the neural promoters directed cell-type-specific transgene expression in the EAE-affected spinal cord. Activity of the neural promoters in and around the inflammatory loci was analyzed based on both cellular morphology of the transduced cells and neuron and glial marker co-localization. The number

Figure 4. Immunohistochemical analysis of promoter activity in the liver, heart, skeletal muscle, and spleen reveals low peripheral activity of the neural promoters compared to the ubiquitous CAG promoter

(A) Representative images revealed strong liver promoter activity for the CAG and gfa2 promoters, whereas the other neural promoters were hardly active in the liver (eGFP [green]). No activity of the neural promoters was detected in the heart, skeletal muscle, and spleen. Scanner settings were kept constant for each analyzed tissue type, and the fluorescence intensity of the images was matched between groups. (B) Quantification of eGFP-expressing cells demonstrated that the CAG and gfa2 promoters directed transgene expression in 61.7% and 17.3% of the liver cells. All other neural promoters revealed liver transduction in less than 1.0% of the liver cells. Each dot represents data from a single animal. Data are presented as mean (SD). N = 4–5 animals per group. Statistics: Brown-Forsythe and Welch ANOVA test with Dunnett's T3 multiple comparison correction. (C–E) Mean eGFP fluorescence (a.u.) in the heart, skeletal muscle, and spleen. None of the neural promoters revealed significant detectable activity in the heart, skeletal muscle, and spleen. In the heart, the CAG promoter was significantly more active compared to the neural promoters. Each dot represents data from a single animal, with data presented as mean (SD). Brown-Forsythe and Welch ANOVA test with Dunnett's T3 multiple comparison correction. N = 4–5 animals per group. Scale bars, 100 μm.

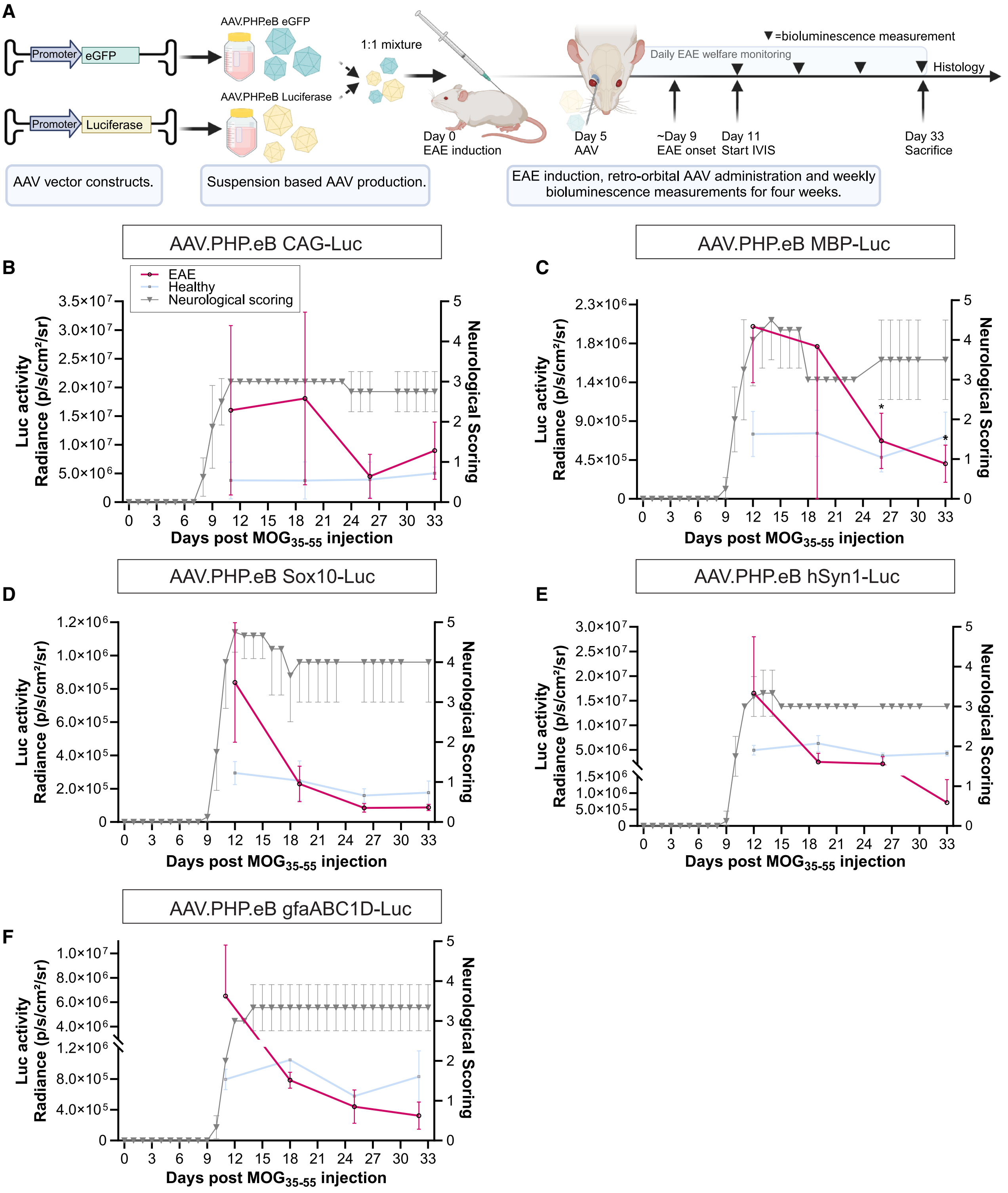

Figure 5. AAV.PHP.eB effectively transduces the CNS with sustained CAG and neural promoter activity in the EAE mouse brain

(A) Schematic overview of the study design. Both AAV.PHP.eB luciferase and eGFP reporter constructs were co-administered via a 1:1 mixture through the retro-orbital sinus, 5 days post-EAE induction with MOG_{35-55}/PTx. Luciferase expression was measured for four subsequent weeks in the brains of EAE mice and plotted together with the

(legend continued on next page)

of transduced cells was quantified within inflammatory lesions and in the surrounding perilesional normal-appearing white matter. These counts were compared to previously established counts of transduced cells in the healthy spinal cord white matter to assess the extent of transgene expression in the EAE-lesioned spinal cords.

In the spinal cords of healthy mice, the CAG promoter was ubiquitously active predominantly in neurons and astrocytes. Under inflammatory EAE conditions, the CAG promoter remained active predominantly in gray matter neurons and astrocytes in the injured spinal cord; however, no transduced cells with distinct oligodendrocyte morphology were detected (Figures 6A and 6B). Both the MBP and Sox10 oligodendroglial promoters remained active and predominantly oligodendrocyte-specific in the lesioned spinal cord (Figures 6A and 6B). Importantly, the use of both MBP and Sox10 promoters resulted in eGFP expression in oligodendrocytes within the inflammation site and the perilesional normal-appearing white matter (Figures 6B, 6D, and 6E). While the absolute number of cells/mm^2 remained relatively high compared to the other neural promoters, the use of the MBP promoter led to a significant 4-fold decrease in the number of cells per area in the lesion ($p < 0.0001$) and a 5-fold decrease in the perilesional white matter compared to the white matter of healthy controls ($p < 0.0001$; Figure 6D). For the Sox10 promoter, no significant difference in the number of transduced cells was found between the lesioned area and perilesional white matter compared to the healthy white matter of healthy animals (Figure 6E). Consistent with observations in the healthy CNS, faint neuronal transgene expression and an occasional eGFP-labeled astrocyte was observed in the EAE spinal cord for both the MBP and Sox10 promoters. Neurons were selectively targeted in the EAE-lesioned spinal cord using the hSyn1 promoter. Similar to the activity of hSyn1 in the spinal cord of healthy controls, no other eGFP-positive glia or non-neural cells were observed in the EAE-affected spinal cord gray or normal-appearing white matter (Figures 6B and 6F). The hSyn1 promoter combined with the eGFP reporter gene resulted in labeling of axons in the white matter (Figures 6A and 6B). In the EAE-lesioned spinal cord, eGFP-labeled axons penetrate the lesion but exhibit signs of axonal degeneration in the white matter near the meninges or tissue edge, where inflammation is present. This degeneration is reflected by a reduction in eGFP signal deeper within the lesion, suggesting loss or fragmentation of axonal structures (Figures 6B and S7). Finally, histological analysis of the lesioned spinal cord showed that the gfaABC1D promoter selectively directed transgene expression in astrocytes in EAE mice. Similar to the healthy spinal cord, there was a high abundance of eGFP-expressing protoplasmic astrocytes in the gray matter and elongated fibrous astrocytes in the normal-appearing white matter. For gfaABC1D, the average number of eGFP-expressing astrocytes/mm^2 did not differ in the inflamed compared to the healthy spinal cord (Figure 6G). Taken together, intravenously administered AAV.PHP.eB combined with the neural promoters enable robust and cell-type-targeted expression in neurons, astrocytes, and oligodendroglia in the EAE mouse model for MS.

DISCUSSION

In this article, a minimally invasive gene therapy method was developed for MS, using EAE, a widely employed MS animal model. To this end, the promoter activity of five neural promoters (MBP, Sox10, hSyn1, gfa2, and gfaABC1D) and one ubiquitous promoter (CAG) was systematically compared in healthy mice and in the EAE mouse model of MS following intravenously delivery via AAV.PHP.eB. Although AAV.PHP.eB cannot cross the BBB in primates, it remains the gold standard for efficiently transducing neurons, astrocytes, and oligodendrocytes in mice. Recently developed BBB-crossing capsids have shown efficacy in primates and exhibit reduced liver tropism. However, these capsids appear to have a shifted tropism favoring neuronal transduction.[41] Therefore, since we aimed to evaluate the cell-type specificity of neural promoters, AAV.PHP.eB was the most logical choice for this study.

A unique aspect of this study was the longitudinal *in vivo* whole-body monitoring of neural promoter activity, paired with detailed histological analysis of their cell-specific activity in several CNS white and gray matter regions, as well as in peripheral organs, all within the same animal. The main findings of this study are as follows: (1) the CAG and neural promoters directed robust, long-term, and stable transgene overexpression in the brains of both healthy and EAE mice; (2) the CAG promoter exhibited strong region-dependent transduction of different cell types with predominant neuronal expression in the cerebral cortex, preferential expression in astrocytes in the brain stem, and approximately equal numbers of neurons and astrocytes in the spinal cord; (3) all neural promoters exhibited a high degree of cellular specificity in the CNS of healthy mice as well as in the lesioned spinal cord of EAE-affected mice. The MBP and Sox10 promoters displayed extensive labeling of oligodendrocytes both around and within inflammatory lesions; and (4) the neural promoters were several orders of magnitude less active in the liver and heart compared to the general CAG promoter. Except for the gfa2 promoter, all neural promoters were minimally active in the liver. None of the promoters was active in skeletal muscle and

previously obtained bioluminescence data in healthy mice (see Figure 1). (B) The CAG promoter remained active in EAE mice, with bioluminescence levels declining after the first 2 weeks to levels comparable to the healthy controls. Notably, the early peak in bioluminescence during EAE likely resulted from increased BBB permeability, facilitating greater luciferin entry into the CNS rather than increased promoter activity.[39,40] $N = 3$ healthy controls; $N = 4$ EAE. (C) The MBP promoter revealed a peak in bioluminescence that coincided with the summit of early EAE symptoms around day 15. After the first 2 weeks, the bioluminescence signal reduced significantly and the expression profile became comparable to the previously measured healthy mice. $N = 4$ EAE mice. Data are presented as mean (SD). (D–F) The Sox10 promoter exhibited luciferase activity that coincided with early EAE symptoms. Likewise, both the hSyn1 and gfaABC1D promoters produced bioluminescence peaks that coincided with early EAE progression after which the brain-associated bioluminescence signals became comparable to those observed earlier in healthy controls. $N = 3$ EAE mice per group. Data are presented as mean (SD).

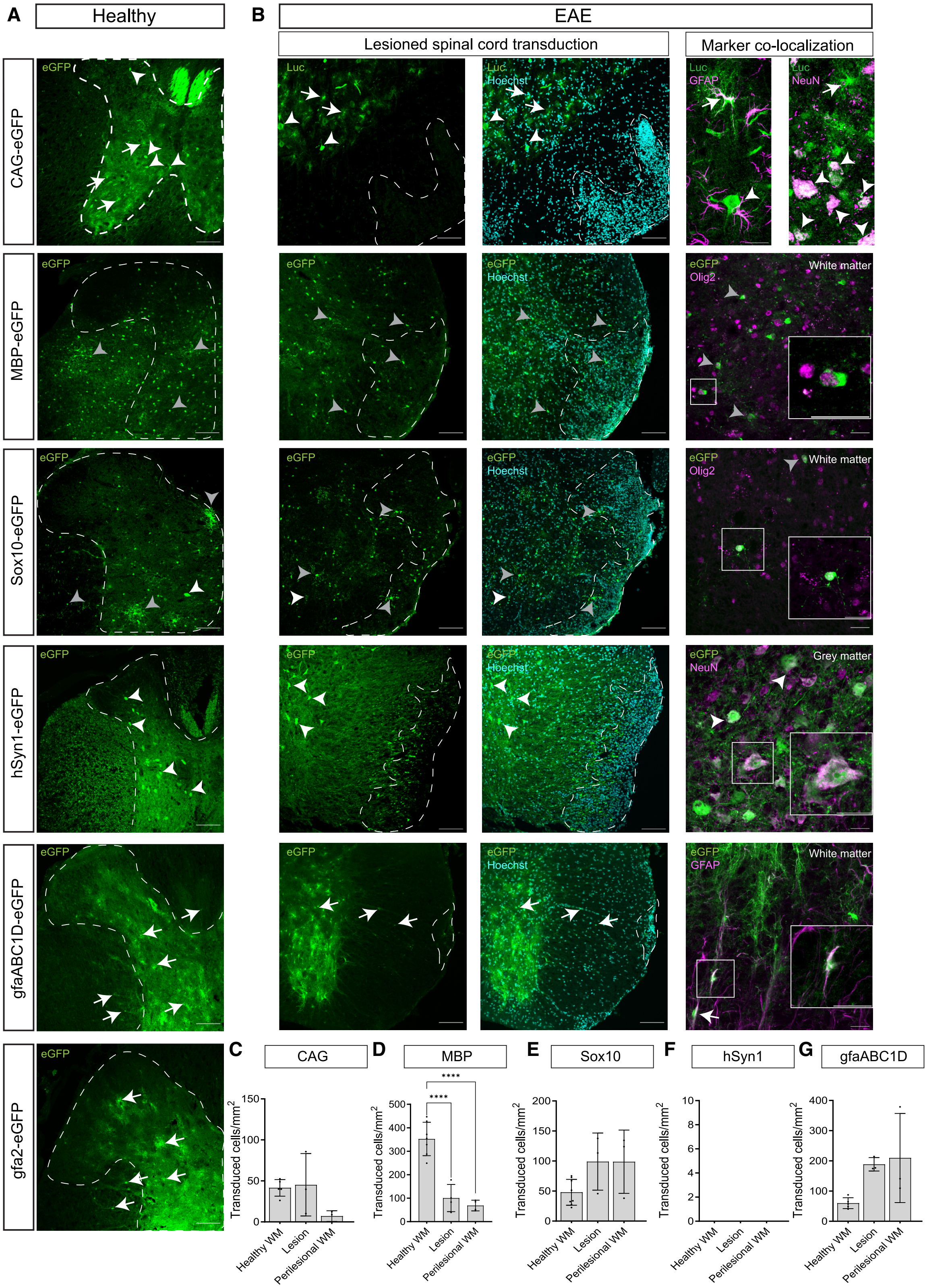

(legend on next page)

the spleen. The use of minimally invasive AAV-based gene transfer in combination with these promoters holds promise for targeting therapeutic genes to specific cell types in the multifocally damaged CNS of EAE mice.

Whole-body, longitudinal *in vivo* imaging in healthy mice demonstrated that the CAG, MBP, Sox10, hSyn1, gfa2, and gfaABC1D promoters directed sustained transgene expression in the brain for 10 weeks. In the brain, the gfa2 promoter exhibited significantly higher bioluminescence compared to the gfaABC1D promoter. This contrasts with findings by Lee et al.,[23] who used transgenic mice with these promoters and showed that the gfaABC1D is 2-fold more active than gfa2 promoter based on β-galactosidase assays of brain extracts. This difference may reflect the tighter epigenetic regulation typically associated with promoters in the genome, whereas episomal nuclear DNA elements such as AAV vectors are generally subject to less stringent epigenetic control.[42,43] Whole-body bioluminescence imaging and histology in EAE mice revealed that AAV.PHP.eB traverses the damaged BBB and effectively transduces CNS cells in both normal-appearing and lesioned spinal cord tissue, with sustained CAG and neural promoter activity throughout the course of EAE. In EAE mice, a peak in bioluminescence coincided with the early stages of the disease. This is most likely due to increased permeability of the BBB resulting in increased levels of luciferin in the CNS, rather than reflecting a temporal increase in promoter activity induced by EAE pathology.[39,40] In both EAE and healthy mice, the bioluminescence signal for all promoters gradually declined over time, which may be due to epigenetic silencing in a subset of the transduced cells or to the gradual loss of vector DNA in slowly proliferating cell populations.[43–46] Although our reporter expression declined over the course of 10 weeks, an approved AAV9-based gene therapy for Spinal Muscular Atrophy type I using the ubiquitous CBA promoter, a variant of the CAG promoter, has shown lasting expression with demonstrated efficacy for over 7.5 years.[47,48]

Since therapeutic protein expression outside the CNS is undesirable, the activity of the neural promoters and CAG in peripheral organs was investigated. Importantly, histology confirmed high activity of the CAG promoter in both the liver and heart, high liver activity for gfa2, and minimal liver activity for the hSyn1, MBP, Sox10, and gfaABC1D promoters. These findings align with a recent study by Finneran et al.,[49] who also reported robust liver and heart expression with the CAG promoter and negligible liver, heart, and muscle activity for the hSyn1 promoter when systemically delivered with AAV.PHP.eB.[49] Systemic AAV delivery is known to result in substantial off-target transduction, particularly in the liver,[50] which may lead to elevated ALT/AST levels and potential long-term liver damage. While we did not assess liver toxicity in this study, the development of next-generation BBB-crossing capsids with reduced liver tropism, such as AAV.CAP.B1, AAV.CAP.B10, and AAV.CAP.B22, offers promising avenues to mitigate these concerns in future systemic gene therapy applications.[41] Intracerebrospinal fluid delivery of future AAV therapies presents a promising approach, as it could lower the required therapeutic viral load and minimize the risk of liver toxicity.

Interestingly, the gfaABC1D promoter labeled significantly fewer liver cells than the gfa2 promoter. Liver activity for gfa2 and gfaABC1D astrocyte promoters was expected, as hepatic stellate cells in the liver are known to express endogenous GFAP.[51–53] The reduced liver activity of gfaABC1D could result from the loss of regulatory elements C2 through C6, which may contain liver-associated enhancers that remain present in gfa2. Robust CNS expression, combined with minimal peripheral activity, makes the gfaABC1D promoter suitable for driving astrocyte-selective transgene expression with limited off-target effects in peripheral tissues. Taken together, except for the gfa2 promoter, the neural promoters are well suited for minimally invasive gene therapies that require cell-directed transgene expression, while minimizing peripheral transgene expression, as demonstrated in healthy mice.

The widely used CAG promoter, known to be ubiquitously active in neurons, astrocytes, and oligodendrocytes and peripheral tissue served as a reference to compare the general activity of this ubiquitous promoter with the cell-type specificity of the neural promoters in both healthy and EAE mice.[5,14,54,55] Consistent with the literature on intravenously delivered AAV9-CAG, which has been studied in the healthy spinal cord by Foust et al.,[14] we also showed that the CAG promoter delivered via AAV.PHP.eB drives transgene expression in predominantly neurons and astrocytes in the healthy mouse CNS and the EAE-affected spinal cord. Interestingly, our results reveal that the CAG promoter is predominantly active in cortical neurons and shows a high preference for astrocytes in the brain stem,

Figure 6. Cell-type-specific promoter activity in the spinal cord in healthy and inflammatory EAE conditions

(A and B) Representative images of transduced cells in healthy controls (left column) and EAE-lesioned spinal cords (eGFP or luciferase [green]; Hoechst [cyan]; cell-type marker [magenta]). Microscope settings for intensity are not equal between experimental groups to improve visualization of transduced cells and co-localizations. In EAE, the CAG promoter remained ubiquitously active in predominantly neurons (arrowhead, co-localization with NeuN) and astrocytes (arrow, co-localization with GFAP). The MBP and Sox10 promoters remained oligodendroglial-specific (gray arrowhead; co-localization with Olig2) and resulted in eGFP-expressing oligodendrocytes around and within inflammatory lesions (dotted line, based on Hoechst). The hSyn1 promoter restricted eGFP expression to neurons (arrowhead, co-localization with NeuN), and eGFP-labeled axons entered the EAE lesions and showed signs of axonal degeneration (zoom in Figure S7). The gfaABC1D promoter selectively labeled astrocytes in the gray and normal-appearing white matter (arrow, co-localization with GFAP). Scale bars 20× magnification, 100 μm; Scale bars 64× magnification (EAE marker co-localization), 25 μm. (C–G) The average number of transduced cells/mm^2 in EAE lesions, the normal-appearing perilesional white matter and the healthy white matter of wild-type controls revealed MBP and Sox10 promoter activity in oligodendrocytes within and around inflammatory lesions. The number of eGFP-labeled astrocytes with gfaABC1D was not altered in EAE compared to healthy controls. In the hSyn1 group, no transduced cells were identified in the spinal cord white matter of either healthy or EAE-affected mice. Healthy control data were previously presented in Figure 3H. Each dot represents data from a single animal. Data are presented as mean (SD). *N* = 3–8 animals per group.

indicating that the CAG promoter may not be as universally active in different cell types as previously assumed. It appears that the activity of the CAG promoter is region-dependent within the CNS. Previous work from others also revealed strong activity of the CAG promoter in cortical and striatal neurons with intraparenchymal administered AAV1-CAG and AAV8-CAG.[54,56] Therefore, the observed preference for cortical neurons appears to not be affected by the route of AAV administration. Others demonstrated predominant astrocyte preference for CAG in the cortex opposed to neurons, despite using the same delivery route, dosage, and AAV.PHP.eB capsid.[5] The observed differences in regional CAG activity across studies could also be attributed to the specific cortical areas analyzed.

Selective targeting of neurons throughout the CNS gray matter in both healthy and EAE mice can be achieved using the hSyn1 promoter. While previous studies in rodents and non-human primates have likewise demonstrated that the hSyn1 promoter is highly neuron-specific in healthy animals, our study is the first to reveal sustained neuron selective activity of the hSyn1 promoter in the context of the EAE mouse model.[57–65] Notably, in the spinal cord white matter of both healthy and EAE-affected mice, the hSyn1 promoter resulted in eGFP labeling of axons. This highlights the potential of the hSyn1 promoter to drive the expression of neuroprotective or promyelination factors in neurons, allowing their delivery to the lesion site via axonal transport. Taken together, the high degree of neuron specificity of the hSyn1 promoter under neuropathological conditions makes the hSyn1 promoter an attractive promoter for neuron-selective gene therapies in MS.

Astrocytes were selectively targeted throughout the CNS using the gfa2 and the gfaABC1D promoters and, as mentioned previously, in the brain stem using the general CAG promoter. The gfaABC1D promoter remained astrocyte-specific in the EAE-lesioned spinal cord. No significant promoter activity was detected in neurons for either of these promoters. This is in line with earlier studies where these promoters were used following AAV-mediated gene delivery.[56,66–71] Interestingly, although most astrocytes in the adult mouse gray matter do not express endogenous GFAP at levels easily detectable via IHC, many GFAP-negative, but GS-positive, astrocytes still expressed eGFP with both promoters. These results suggest that astrocyte promoter activity in the context of an intravenously delivered AAV vector does not match the *Gfap* gene expression profile. Although GFAP-positive astrocytes were highly abundant in the EAE-affected spinal cord white matter, the gfaABC1D promoter did not result in a noticeable increase in transgene expression in astrocytes in the spinal cord of EAE mice compared to the healthy spinal cord. In contrast to our findings in the EAE model, the gfa2 promoter has previously been shown to closely follow endogenous GFAP levels in transgenic mice following stab wound injury in the cerebral cortex.[72] However, in EAE, the increased GFAP immunoreactivity likely reflects not only elevated GFAP protein levels due to astrogliosis but also enhanced epitope accessibility, which can amplify the immunohistochemical signal.[73,74] Although the gfa2 and gfaABC1D promoters are both derived from the *GFAP* gene, we observed transgene expression in astrocytes that were GFAP-negative by IHC. Several factors may account for this: first, the promoter fragments used in AAV vectors differ from the endogenous *Gfap* regulatory region not only in genomic context but also in regulatory content, such as the absence of intronic and/or distal enhancers and silencers, as well as changes in the arrangement or three-dimensional positioning of transcription factor binding sites. These differences could modify promoter specificity, potentially broadening promoter activity to include astrocytes that would not normally express the endogenous *Gfap* gene under similar conditions; second, while the endogenous *Gfap* gene is often transcriptionally silenced under homeostatic conditions due to chromatin compaction and repressive chromatin marks, the AAV genome remains episomal and more accessible to transcription factors, potentially allowing expression in astrocytes with low or undetectable GFAP levels; and third, the use of stable reporter proteins such as eGFP or luciferase may lead to sustained signal even if promoter activity is intermittent or transient. This may explain why we did not observe a strict correspondence between GFAP staining and promoter-driven reporter expression. Given its small size and astrocyte-selective expression, the gfaABC1D promoter is well suited for driving astrocyte-targeted therapy development in a mouse model of MS. Since astrocytes are a component of the glial scar in MS lesions, overexpressing therapeutic factors in astrocytes could be a promising strategy to promote the secretion of therapeutic molecules at the site of inflammation. Others also demonstrated strong translational potential of gfaABC1D, as it drives astrocyte-restricted expression in the globus pallidus and striatum following intraparenchymal AAV5 administration in rhesus macaques.[22] Taken together, these results highlight the potential use of gfaABC1D for astrocyte-targeted therapies in human CNS diseases, where widespread astrocyte-specific expression is crucial for therapeutic efficacy.

The MBP and Sox10 promoters direct selective overexpression of transgenes in oligodendrocytes in the healthy CNS and under neuroinflammatory conditions in EAE. Importantly, both the MBP and Sox10 promoters selectively labeled oligodendrocytes within and around inflammatory lesions in EAE. This targeted transgene expression in oligodendrocytes, especially near or at lesion sites, is highly relevant for future gene therapies aimed at multifocal EAE lesions, as these promoters enable the delivery of therapeutic proteins directly to myelinating cells in proximity to demyelinating lesions. The MBP promoter directed transgene expression in significantly more oligodendrocytes compared to the Sox10 promoter. A possible explanation for this could be that in adult mouse CNS, the endogenous Sox10-MCS5/U2 enhancer activity is repressed in mature oligodendrocytes.[75] Although the Sox10 promoter has been shown to drive expression in human and rodent primary oligodendrocytes and OPCs, its use in the adult mouse CNS was limited, with only occasional detection of eGFP-expressing OPCs and a relatively low number of transduced oligodendrocytes. To our knowledge, there is no existing literature on the tropism of AAV.PHP.eB for OPCs. Therefore, our results may reflect the limited ability of the AAV.PHP.eB capsid to transduce OPCs in the adult mouse CNS, rather than an absence of promoter activity in these

Table 1. Overview of expression cassettes of each viral vector construct

Construct	Promoter size (bp)	Total size (bp)
ITR2-CAG-NV5Luc2-bGHpolyA-ITR2	859	3,359
ITR2-CAG-eGFP-bGHpolyA-ITR2		2,472
ITR2-gfa2-NV5Luc2-bGHpolyA-ITR2	2,178	4,674
ITR2-gfa2-eGFP-bGHpolyA-ITR2		3,781
ITR2-gfaABC1D-NV5Luc2-bGHpolyA-ITR2	583	3,099
ITR2-gfaABC1D-eGFP-bGHpolyA-ITR2		2,206
ITR2-MBP-NV5Luc2-bGHpolyA-ITR2	1,963	4,460
ITR2-MBP-eGFP-bGHpolyA-ITR2		3,567
ITR2-Sox10-NV5Luc2-bGHpolyA-ITR2	800	3,322
ITR2-Sox10-eGFP-bGHpolyA-ITR2		2,429
ITR2-hSyn1-NV5Luc2-bGHpolyA-ITR2	469	2,997
ITR2-hSyn1-eGFP-bGHpolyA-ITR2		2,104

Construct size (bp) is the number of bp including the ITRs.

cells. However, it is also possible that transduced OPCs were not detected after 10 weeks of expression due to their high proliferation rate, which could lead to dilution of episomal DNA during mitosis, as well as their maturation into oligodendrocytes that no longer express PDGFRα.[76,77] As expected, both the MBP and Sox10 promoters resulted in occasional faint labeling of neurons and astrocytes. Although neuronal and astrocyte activity was not previously reported for the Sox10 promoter, weak MBP promoter activity in neurons and occasional astrocyte labeling is supported by von Jonquieres and colleagues,[34] who found age-dependent non-oligodendrocyte activity for the MBP promoter. While other studies have reported substantial neuronal labeling with the MBP promoter, our findings reveal minimal promoter activity in neurons. This discrepancy is likely due to the dilution effect associated with delivery into larger fluid compartments, such as systemic administration, leading to weaker transduction compared to targeted injection into smaller fluid compartments, like the brain parenchyma or cerebrospinal fluid.[78,79] It is important to note that, although positive neurons and astrocytes were detected for both oligodendrocyte promoters, off-target expression in this small number of non-oligodendroglial cells is unlikely to impact the development of a gene therapy for MS. However, the required cell-type specificity and the potential negative effects of off-target expression depend largely on the therapeutic factor used and should be assessed through dose-response studies. Additionally, the usage of AAV capsids with preferential oligodendrocyte tropism could further minimize off-target activity for Sox10 and MBP.[80] Taken together, our results demonstrate that the MBP and Sox10 promoters are suitable for targeting transgene expression in oligodendrocytes, despite some off-target activity in neurons and astrocytes, and would be especially effective for delivering therapeutic proteins near and within inflammatory EAE lesions.

Conclusion

To improve therapeutic success of systemic gene therapies for dynamic, multifocal disorders like MS, it is important that therapeutic genes are expressed selectively in the cell types associated with the disease pathology in the CNS. In this study, we reveal that neurons, astrocytes, and oligodendrocytes can be selectively targeted throughout the CNS with sustained cell-type specificity in the EAE-lesioned spinal cord. In healthy mice, we demonstrated minimal peripheral activity for the neural promoters, except for gfa2. This study is an important first step toward the development of a minimally invasive gene therapy to stimulate myelin and lesion repair in MS.

MATERIALS AND METHODS

Recombinant adeno-associated virus vectors

Recombinant AAV vector plasmids were produced by using standard molecular cloning techniques. As starting construct, the AAV2 ITR from Vector BioLabs (Vector BioLabs, Philadelphia, PA, USA) was used since this vector is modified to significantly reduce the frequency of ITR recombination events. A multiple cloning site was inserted in the vector backbone to facilitate the further cloning procedure. The promoters used in this study were the CAG promoter that consists of the cytomegalovirus (CMV) immediate-early enhancer, the chicken beta-actin promoter, and a hybrid intron derived from the rabbit beta-globin gene (CAG; 859 bp), human glial fibrillary acidic protein 2 (gfa2[72,81]; 2178 bp), the shorter modified gfa2 version (gfaABC1D[23]; 583 bp), *Homo sapiens* synapsin 1 (hSyn1[54,82,83]; 469 bp), *Mus musculus* myelin basic protein promoter (MBP[34]; 1963 bp), and the *Mus musculus* Sox10[21] promoter consisting of the phylogenetically highly conserved enhancer element 5 (MCS5) upstream of the c-fos basal promoter (800 bp; Addgene cat#115783). The coding sequence of N-terminally V5-labeled firefly luciferase 2 (NV5luc2) was inserted into the multiple cloning site. Thereafter CAG, gfa2, gfaABC1D, hSyn1, MBP, and Sox10 were removed from their respective donor plasmids and inserted upstream of NV5luc2 in the vector backbone. For the eGFP plasmids, N5Vluc2 was enzymatically removed and the coding sequence of eGFP was inserted downstream of each promoter element. Both eGFP and NV5luc2 contained the Kozak sequence (5′-GCCAC CATGG-3′). Each construct contained the bovine growth hormone polyadenylation signal (bGH-polyA). An overview of the expression casettes used is shown in Table 1.

Suspension-based recombinant adeno-associated virus production

Recombinant, single-stranded AAV vectors were produced using our suspension-based production platform.[84] VP293 cells at a density of $\sim 2.5 \times 10^{11}$ cells/mL were transfected with transfer, helper, and capsid plasmids using PEI-MAX (Polysciences); 48 to 72 h post-transfection, the suspension cells were lysed, and total protein was precipitated overnight at 4°C using Polyethylene glycol 8000 (PEG_{8000}). AAV was further purified via iodixanol purification using an ultracentrifuge. Finally, iodixanol was buffer exchanged for PBS (no magnesium, no chloride (−/−); 5.0% sucrose). AAV.PHP.eB stocks were stored at 4°C. AAV.PHP.eB titers were determined through quantitative PCR (qPCR). The bGH-polyA was used as primer binding target: bGH-polyA forward primer sequence: 5′-GCCTTCTAGTTGCCAGCCAT-3′; bGH-polyA reverse primer

sequence: 5′-GGCACCTTCCAGGGTCAAG-3′. Undigested, circular plasmid was utilized as a standard. qPCR was performed using SYBR Green Dye (SYBR Green PCR Master Mix, Applied Biosystems, Foster City, CA, USA), according to the manufacturer's protocol.

Animals

A total of 55 female C57Bl/6N mice (B6N-Tyrc-Brd/BrdCrCrl; Charles River Laboratories, Italy) and 7 female C57BL/6J mice (Janvier Labs, St. Berthevin, France) were used. These albino mice are a spontaneous co-isogenic C57BL/6N strain harboring a mutation in the tyrosinase gene, which prevents the formation of melanin. We utilized albino B6 mice since previous bioluminescence experiments using regular black C57Bl/6J mice frequently resulted in randomly distributed spots of collagen deposition and skin pigmentation at the shaved skin, which significantly affected the bioluminescence readout parameter. This phenomenon was also reported by others.[24] All animals were between 6 and 10 weeks old at the start of the experiment, with a body weight ranging between 18 and 30 g. Mice were housed under standard conditions with *ad libitum* access to food and water. Due to technical challenges related to freezing and perfusion, spinal cord analysis was not feasible for some mice in the MBP and Sox10 groups. To ensure sufficient data, three additional mice per group were administered AAV and sacrificed after 10 weeks, resulting in N = 3–8 for some analyses. All experimental procedures were performed in accordance with the European guidelines for the Care and Use of Laboratory Animals (86/609/EEC) and were approved by the animal committee of the Royal Netherlands Academy of Arts and Sciences.

Intravenous adeno-associated virus administration

All mice received 5 x 10^{11} genomic copies (gc) of each promoter-reporter vector through the retro-orbital sinus, totaling 1 x 10^{12} gc/mouse when two vectors were administered together. Thirty-gauge insulin needles (BD micro or ultra-fine insulin syringes) were used, and the total injection volume did not exceed 130 μL per mouse. All mice were deeply anesthetized with isoflurane (Isoflo, Abbott, the Netherlands) (induction 5%; maintenance 1.5%–3.0%). After anesthetic induction, the scalp was fixed dorsal and ventral to the eye, resulting in the eye bulb to partially protrude the eye from the eye socket. The syringe was placed in the retro-bulbar space pointing the syringe, with the bevel of the needle down to avoid damaging the eye bulb.

In vivo IVIS bioluminescence imaging

All mice were deeply anesthetized in an induction chamber with 5% isoflurane (Isoflo, Abbott, the Netherlands). While still under anesthesia, hair was trimmed from the scalp, neck, and back (Aesculap Trimmer Isis GT421). Mice were placed on a heated platform (37°C) in the IVIS 200 imaging system (Caliper Live Sciences, Hopkinton, MA, USA) (isoflurane maintenance 1.5%–3.0%). Two hundred microliters of luciferin D-salt solution (375 mg/mL phosphate buffered 0.9% saline; Regis Technologies, Morton Grove, IL, USA) was injected intraperitoneally. Since time is needed for luciferin to homogeneously reach all tissues, the average radiance (p/s/cm^2/sr) was measured until no further increase in bioluminescence signal was observed anymore. The maximum radiance measured during the *in vivo* imaging system (IVIS) session was taken as the readout parameter. Exposure time was set to auto-exposure to automatically set the exposure time, f/stop, and binning to keep the signal within an optimal range for quantification.

Perfusion and tissue preparation

Mice were euthanized by injecting an overdose of Pentobarbital (100 mg/kg diluted in 0.9% saline, Amsterdam Medical Center Apothecary) intraperitoneally (i.p.). Toe pinch reflex was tested to verify the animal was fully sedated. An incision was made using a surgical scissor (Sharp-Blunt, 14001-12, Fine Science Tools) to open the diaphragm and reach the atrium and the right ventricle. The needle attached to the pump was inserted in the apex of the heart, and a cut was made in the right atrium using surgical scissors (Spring Scissors-6 mm Cutting Edge, Fine Science Tools). First, the circulatory system was rinsed with 0.9% saline after which the mouse was perfused with 4% Paraformaldehyde (PFA) in 0.1 M phosphate buffer. After perfusion, dissected tissue was placed in an incubation vial to post-fixate in 4% PFA for 2 hours at 4°C. After the 4% PFA incubation, the brain was transferred to a new incubation vial containing 25% sucrose phosphate buffer (PB) overnight (O/N). Tissue was embedded in Tissue-Tek O.C.T. Compound (Sakura Finetek, USA) and snap frozen by submerging the O.C.T. embedded tissue in 2-methylbutane in a container cooled by dry ice.

Immunohistochemistry

CAG-driven luciferase expression yielded excellent antibody labeling using the 60CCR2029RAF luciferase antibody (1:100; Fitzgerald Industries International). However, pilot studies showed that the weaker neural promoters did not produce sufficient luciferase staining. Therefore, mice received a 1:1 mixture of promoter-driven luciferase and eGFP reporter vectors. All tissues used were sectioned at 20 μm using the Leica CM3050S at a chamber temperature of approximately −21°C. For sectioning brain tissue, we sectioned approximately 1,200 μm, starting from the medial longitudinal fissure in one series of 12 Superfrost Plus adhesion microscope slides (Epredia, Cat. No. J1800AMNZ). For IHC, the sections were rinsed with tris-buffered saline (TBS), blocked for 1 hour at room temperature (RT) with blocking buffer (5% fetal bovine serum in TBS with 0.1% Triton X-100(v/v)) and incubated overnight at 4°C with primary antibodies (GFP, NeuN, Iba1, GFAP, GS, Olig2, and PDGFRα; details below) diluted in blocking buffer. The following day, sections were rinsed three times in TBS and incubated for 2 hours with fluorescent-labeled secondary antibodies diluted in blocking buffer. Following a final wash in TBS supplemented with Hoechst, the sections were rinsed in miliQ H_2O, air dried, and cover slipped (24 × 60mm #1, Menzel-Gläzer) using Mowiol. The following antibodies were used: GFP (Chicken, 1:400, AB16901), detected with Donkey anti-Chicken 488 (1:800); NeuN (Mouse, 1:500, MAB377), detected with Donkey anti-Mouse Cy3 (1:800); Iba1 (Rabbit, 1:500, ab178847), detected with Donkey anti-Rabbit Cy5 (1:800); GFAP (Cy3) (Mouse, 1:400, C9205); GS (Mouse, 1:500, MAB302), detected with Goat anti-IgG2A AF647 (1:800); Olig2

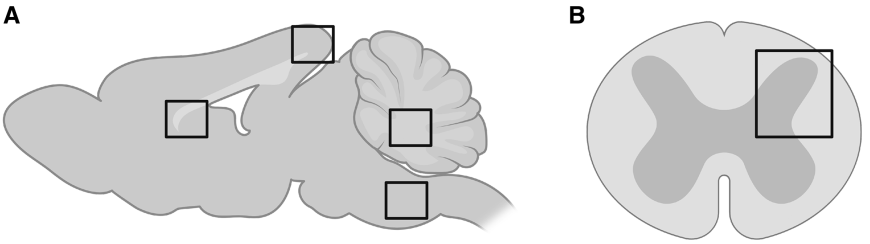

Figure 7. Schematic overview of the nervous system areas for quantification

(A) Imaged regions in the brain included the cortex (rsc), corpus callosum, cerebellar white matter, and brain stem (grn), all imaged at 20× (dry) magnification (single z stacks). In the cortex and brain stem, all transduced cells within the confocal image were analyzed for co-localization with the cell-type markers. For the corpus callosum and cerebellum, only the white matter was analyzed and not the surrounding gray matter or the deep cerebellar nuclei. (B) Imaged region of the spinal cord gray matter and white matter imaged at 20× (dry) confocal magnification (single z stacks).

(Mouse, 1:500, MABN50), detected with Goat anti-IgG2A AF647 (1:800); PDGFRα (Rabbit, 1:500, ab203491), detected with Donkey anti-Rabbit Cy5 or Cy3 (1:800); and Luciferase (Rabbit, 1:100, 60CCR2029RAF), detected with Donkey anti-Rabbit 488 (1:800).

Imaging of brain, spinal cord, and peripheral tissue

Immunofluorescent double-labeling was performed on brain and spinal cord tissue to analyze CNS cell-type specificity and efficiency of the CAG, MBP, Sox10, hSyn1, gfa2, and gfaABC1D promoters. Primary and secondary antibody combinations and concentrations were used as described above. Single Z-plane images were taken of the brain, including the retrosplenial cortex (rsc), rostral body of the corpus callosum, cerebellar white matter, and the gigantocellular reticular nucleus (grn) in the medulla oblongata. (Figure 7A). A total of five coupes per brain region per co-labeling per animal were imaged using the Leica TCS SP5 confocal microscope at 20× (dry) magnification. Representative z stack confocal images of all co-labeling for all markers in the cortex were made at 63× magnification (oil); For 20× and 63× magnification images shown, the contrast was optimized with ImageJ to improve visual clarity (National Institutes of Health, Bethesda, MD, USA, version 1.53).

Similar to brain tissue, spinal cord tissue was co-labeled using the immunohistochemical markers described in the immunohistochemistry section. Single Z-plane confocal images were taken from the dorsal horn and white matter of the spinal cord at 20× dry magnification (Figure 7B). A minimum of three confocal images were analyzed per co-immunohistochemical staining per mouse per group.

For liver, heart, spleen, and quadriceps femoris tissue, a ZEISS Axioscan 7 was used. Tissue was scanned at a 10× magnification for the excitation/emission of Alexa488 for eGFP and UV for Hoechst. Three technical replicates were used per quantification. For skeletal muscle of the Sox10 group, we could only analyze two biological replicates with three technical replicates due to either loss and/or inadequate quality of the sections.

Quantification of immunofluorescence to deduce promoter specificity

To assess the specificity and transduction efficiency of each promoter element, all eGFP-immunoreactive cells were quantified using the Qupath software (version 0.4.2.). Mean co-localization was based on at least three animals per staining per region. To determine the transduction efficiency of AAV.PHP.eB promoter-reporter combinations in the CNS, all eGFP-positive cells co-localizing with cell-type-specific markers were counted manually using the annotation tool in QuPath. Cells expressing eGFP were included when the fluorescence level was at least three times higher than the background. For images taken of the cerebellar white matter and corpus callosum, we annotated and analyzed solely white matter tissue, excluding the gray matter. Confocal images were excluded from analysis if repeated IHC attempts did not yield consistent and reliable marker visualization. The mean eGFP/marker co-localization was based on a minimum of three animals per promoter per marker per region. To calculate the cell-type specificity of each promoter in the analyzed CNS regions, the percentage of doubled-labeled cells was calculated for each animal as the total number of eGFP-positive cells that also expressed the cell-specific marker of interest divided by the total number of eGFP-immunoreactive cells across all analyzed images for that animal. The transduction efficiency in the brain stem was determined as the total number of eGFP-positive cells that co-localized with the cell-specific marker over the total number of cells that were immunoreactive for the marker.

For quantification of eGFP reporter expression for all promoters in the liver, we used automatic cell detection using QuPath based on Hoechst nucleic acid staining. Liver transduction data from four control animals that received empty AAV.PHP.eB were added to confirm the absence of eGFP-positive liver cells in absence of eGFP expression. Thereafter, we automatically determined the percentage of detected nuclei that were also immunoreactive for eGFP to approximate the percentage of transduced cells. Since (cardiac) muscle tissue is multinucleated and single cells were challenging to detect in the red and white pulp of the spleen, a different quantification methodology was applied. For cardiac muscle, skeletal muscle, and spleen, a large region of interest was selected, excluding holes, and the mean eGFP fluorescence was measured. We aimed for three technical replicates per organ per group.

Acute experimental autoimmune encephalomyelitis induction and clinical evaluation

EAE mice (albino C57BL/6N: B6N-Tyrc-Brd/BrdCrCrl; Charles River Laboratories, Italy) and C57BL/6J mice (Janvier Labs, St.

Berthevin, France) were immunized with the Hooke kit (EK-2110, Hooke Laboratories, USA) according to the manufacturer's instructions. All mice used in this article were between 6 and 10 weeks old and were 18–30 g at the start of the experiment. Mice were anesthetized by 5% isoflurane induction (Isoflo, Abbott, the Netherlands) and were transferred to a nose cone delivering 1.5% isoflurane maintenance, aiming to minimize stress. Heavily anesthetized mice received two subcutaneous injections of 100 μL MOG_{35-55} in CFA on the midline of the upper back and on the midline of the lower back. Two hours post-injection (p.i.), the mice received 100 μL 200 ng/μL Pertussis Toxin in glycerol buffer (PTx, lot #1015 Hooke Laboratories). Twenty-four hours p.i., mice received a second dosage of PTx. Mice received daily wet food pallets, hydrogel, and nutrient paste. Mice were weighted and evaluated daily for neurological signs of EAE using the 10-score system: 0, no clinical disease; 0.5, paralysis of the tail tip; 1, complete tail paralysis; 2, hindlimb weakness; 3, severe hindlimb weakness; 4, unilateral hindlimb paralysis; 5, bilateral hindlimb paralysis; 6, paralysis of the complete abdomen; 7, partial forelimb weakness; 8, moribund; and 9, death.

Statistics

All statistics and graphs were made using the GraphPad Prism 10 for windows (GraphPad Software, Boston, Massachusetts USA). To test significant differences in IVIS-measured bioluminescence signal between promoters in healthy mouse brains, livers, and the brain/liver ratio, we performed a Brown-Forsythe and Welch ANOVA with Dunnett's T3 multiple comparison analysis because the data were normally distributed based on Q-Q plot analysis but heteroscedastic based on Brown-Forsythe test. To test the significant difference in eGFP counts or fluorescence intensities for liver, heart, skeletal muscle, and spleen, we performed Brown-Forsythe and Welch ANOVA test with Dunnett's T3 multiple comparisons test. Since the histological marker co-localization data were normally distributed (Q-Q plot), heteroscedastic (Brown-Forsythe), and the co-localization contained null values, which prevented use of a Brown-Forsythe and Welch ANOVA, a non-parametric Kruskal-Wallis test was used. In EAE mice, we examined whether the bioluminescence signal observed in the first week was significantly different compared to the other weeks. For this, we performed a repeated measures one-way ANOVA with Geisser-Greenhouse correction and Dunnett's multiple comparison test for MBP, Sox10, CAG, and gfaABC1D. Due to a missing value related to a deceased animal, a mixed effects analysis with Geisser-Greenhouse correction and Dunnett's multiple comparison test was performed for hSyn1 with Dunnett's multiple comparison test. To assess differences in the density of transduced cells per mm^2 between healthy spinal cord white matter and EAE-affected white matter, an ordinary one-way ANOVA with Tukey's post hoc correction for multiple comparisons was used. For all figures, adjusted p values are represented as follows: $*p < 0.05$; $**p < 0.01$; $***p < 0.001$; $****p < 0.0001$.

DATA AVAILABILITY

Data supporting the findings of this study are available upon request from the corresponding author.

ACKNOWLEDGMENTS

The authors would like to thank Roeland Lokhorst for assisting with confocal microscopy, Eline Feenstra for helping in the design of the graphical abstract, and Aditi Jagannathan for assisting in the proofreading of the manuscript. We appreciate Alexander Heimel, PhD, for his guidance on the statistical analysis. Parts of figures in this manuscript were created using BioRender.com. This study was generously funded by the Start2Cure Foundation (project 0-TI-01) and Health Holland (project LSHM22014).

AUTHOR CONTRIBUTIONS

Conceptualization, J.V., F.d.W., and I.H. Investigation, P.J.H.N., M.A.R., R.H., and G.v. Z. Writing the paper, P.J.H.N., J.V., and F.d.W. Supervision, J.V., F.d.W., and I.H.

DECLARATION OF INTERESTS

The authors declare no competing interests.

DECLARATION OF GENERATIVE AI AND AI-ASSISTED TECHNOLOGIES IN THE WRITING PROCESS

During the preparation of this work the author(s) used ChatGPT 4.0 in order to facilitate grammatical accuracy. After using this tool/service, the author(s) reviewed and edited the content as needed and take(s) full responsibility for the content of the publication.

SUPPLEMENTAL INFORMATION

Supplemental information can be found online at https://doi.org/10.1016/j.omtm.2025.101504.

REFERENCES

1. Luchetti, S., Fransen, N.L., van Eden, C.G., Ramaglia, V., Mason, M., and Huitinga, I. (2018). Progressive multiple sclerosis patients show substantial lesion activity that correlates with clinical disease severity and sex: a retrospective autopsy cohort analysis. Acta Neuropathol. *135*, 511–528. https://doi.org/10.1007/s00401-018-1818-y.
2. Castle, M.J., Baltanás, F.C., Kovacs, I., Nagahara, A.H., Barba, D., and Tuszynski, M. H. (2020). Postmortem analysis in a clinical trial of AAV2-NGF gene therapy for Alzheimer's disease identifies a need for improved vector delivery. Hum. Gene Ther. *31*, 415–422. https://doi.org/10.1089/hum.2019.367.
3. Smolders, J., Fransen, N.L., Hsiao, C.C., Hamann, J., and Huitinga, I. (2020). Perivascular tissue resident memory T cells as therapeutic target in multiple sclerosis. Expert Rev. Neurother. *20*, 835–848. https://doi.org/10.1080/14737175.2020.1776609.
4. Deverman, B.E., Ravina, B.M., Bankiewicz, K.S., Paul, S.M., and Sah, D.W.Y. (2018). Gene therapy for neurological disorders: Progress and prospects. Nat. Rev. Drug Discov. *17*, 641–659. https://doi.org/10.1038/nrd.2018.110.
5. Ravindra Kumar, S., Miles, T.F., Chen, X., Brown, D., Dobreva, T., Huang, Q., Ding, X., Luo, Y., Einarsson, P.H., Greenbaum, A., et al. (2020). Multiplexed Cre-dependent selection yields systemic AAVs for targeting distinct brain cell types. Nat. Methods *17*, 541–550. https://doi.org/10.1038/s41592-020-0799-7.
6. Chan, K.Y., Jang, M.J., Yoo, B.B., Greenbaum, A., Ravi, N., Wu, W.L., Sánchez-Guardado, L., Lois, C., Mazmanian, S.K., Deverman, B.E., and Gradinaru, V. (2017). Engineered AAVs for efficient noninvasive gene delivery to the central and peripheral nervous systems. Nat. Neurosci. *20*, 1172–1179. https://doi.org/10.1038/nn.4593.
7. Lyon, A.R., Babalis, D., Morley-Smith, A.C., Hedger, M., Suarez Barrientos, A., Foldes, G., Couch, L.S., Chowdhury, R.A., Tzortzis, K.N., Peters, N.S., et al. (2020). Investigation of the safety and feasibility of AAV1/SERCA2a gene transfer in patients with chronic heart failure supported with a left ventricular assist device – the SERCA-LVAD TRIAL. Gene Ther. *27*, 579–590. https://doi.org/10.1038/s41434-020-0171-7.
8. Hudry, E., and Vandenberghe, L.H. (2019). Therapeutic AAV Gene Transfer to the Nervous System: A Clinical Reality. Neuron (Camb., Mass.) *101*, 839–862. https://doi.org/10.1016/j.neuron.2019.02.017.
9. Gaj, T., Epstein, B.E., and Schaffer, D.V. (2016). Genome engineering using Adeno-associated virus: Basic and clinical research applications. Mol. Ther. *24*, 458–464. https://doi.org/10.1038/mt.2015.151.

10. Nathwani, A.C., Reiss, U.M., Tuddenham, E.G.D., Rosales, C., Chowdary, P., McIntosh, J., Della Peruta, M., Lheriteau, E., Patel, N., Raj, D., et al. (2014). Long-term safety and efficacy of factor IX gene therapy in hemophilia B. N. Engl. J. Med. *371*, 1994–2004. https://doi.org/10.1056/NEJMOA1407309.

11. Ellsworth, J.L., Gingras, J., Smith, L.J., Rubin, H., Seabrook, T.A., Patel, K., Zapata, N., Olivieri, K., O'Callaghan, M., Chlipala, E., et al. (2019). Clade F AAVHSCs cross the blood brain barrier and transduce the central nervous system in addition to peripheral tissues following intravenous administration in nonhuman primates. PLoS One *14*, e0225582. https://doi.org/10.1371/JOURNAL.PONE.0225582.

12. Hocquemiller, M., Giersch, L., Audrain, M., Parker, S., and Cartier, N. (2016). Adeno-Associated Virus-Based Gene Therapy for CNS Diseases. Hum. Gene Ther. *27*, 478–496. https://doi.org/10.1089/HUM.2016.087/ASSET/IMAGES/LARGE/FIGURE1.JPEG.

13. Wang, D., Tai, P.W.L., and Gao, G. (2019). Adeno-associated virus vector as a platform for gene therapy delivery. Nat. Rev. Drug Discov. *18*, 358–378. https://doi.org/10.1038/s41573-019-0012-9.

14. Foust, K.D., Nurre, E., Montgomery, C.L., Hernandez, A., Chan, C.M., and Kaspar, B.K. (2009). Intravascular AAV9 preferentially targets neonatal neurons and adult astrocytes. Nat. Biotechnol. *27*, 59–65. https://doi.org/10.1038/nbt.1515.

15. Deverman, B.E., Pravdo, P.L., Simpson, B.P., Kumar, S.R., Chan, K.Y., Banerjee, A., Wu, W.-L., Yang, B., Huber, N., Pasca, S.P., and Gradinaru, V. (2016). Cre-dependent selection yields AAV variants for widespread gene transfer to the adult brain. Nat. Biotechnol. *34*, 204–209. https://doi.org/10.1038/NBT.3440.

16. Ophir, O., Levy, G., Bar, E., Kimchi Feldhorn, O., Rokach, M., Elad Sfadia, G., and Barak, B. (2023). Deletion of Gtf2i via Systemic Administration of AAV-PHP.eB Virus Increases Social Behavior in a Mouse Model of a Neurodevelopmental Disorder. Biomedicines *11*, 2273. https://doi.org/10.3390/biomedicines11082273.

17. Palfi, A., Chadderton, N., Millington-Ward, S., Post, I., Humphries, P., Kenna, P.F., and Farrar, G.J. (2022). AAV-PHP.eB transduces both the inner and outer retina with high efficacy in mice. Mol. Ther. Methods Clin. Dev. *25*, 236–249. https://doi.org/10.1016/j.omtm.2022.03.016.

18. Mathiesen, S.N., Lock, J.L., Schoderboeck, L., Abraham, W.C., and Hughes, S.M. (2020). CNS Transduction Benefits of AAV-PHP.eB over AAV9 Are Dependent on Administration Route and Mouse Strain. Mol. Ther. Methods Clin. Dev. *19*, 447–458. https://doi.org/10.1016/j.omtm.2020.10.011.

19. O'Carroll, S.J., Cook, W.H., and Young, D. (2020). AAV Targeting of Glial Cell Types in the Central and Peripheral Nervous System and Relevance to Human Gene Therapy. Front. Mol. Neurosci. *13*, 618020. https://doi.org/10.3389/fnmol.2020.618020.

20. Griffin, J.M., Fackelmeier, B., Fong, D.M., Mouravlev, A., Young, D., and O'Carroll, S.J. (2019). Astrocyte-selective AAV gene therapy through the endogenous GFAP promoter results in robust transduction in the rat spinal cord following injury. Gene Ther. *26*, 198–210. https://doi.org/10.1038/s41434-019-0075-6.

21. Pol, S.U., Lang, J.K., O'bara, M.A., Cimato, T.R., Mccallion, A.S., and Sim, F.J. (2013). Sox10-MCS5 enhancer dynamically tracks human oligodendrocyte progenitor fate. Exp. Neurol. *247*, 694–702. https://doi.org/10.1016/j.expneurol.2013.03.010.

22. Heffernan, K.S., Rahman, K., Smith, Y., and Galvan, A. (2022). Characterization of the GfaABC1D promoter to selectively target astrocytes in the rhesus macaque brain. J. Neurosci. Methods *372*, 109530. https://doi.org/10.1016/J.JNEUMETH.2022.109530.

23. Lee, Y., Messing, A., Su, M., and Brenner, M. (2008). GFAP promoter elements required for region-specific and astrocyte-specific expression. GLIA (New York, N. Y.) *56*, 481–493. https://doi.org/10.1002/glia.20622.

24. Curtis, A., Calabro, K., Galarneau, J.R., Bigio, I.J., and Krucker, T. (2011). Temporal variations of skin pigmentation in C57Bl/6 mice affect optical bioluminescence quantitation. Mol. Imaging Biol. *13*, 1114–1123. https://doi.org/10.1007/s11307-010-0440-8.

25. Ben Haim, L., Schirmer, L., Zulji, A., Sabeur, K., Tiret, B., Ribon, M., Chang, S., Lamers, W.H., Boillée, S., Chaumeil, M.M., and Rowitch, D.H. (2021). Evidence for glutamine synthetase function in mouse spinal cord oligodendrocytes. GLIA (New York, N. Y.) *69*, 2812–2827. https://doi.org/10.1002/GLIA.24071.

26. Cammer, W. (1990). Glutamine synthetase in the central nervous system is not confined to astrocytes. J. Neuroimmunol. *26*, 173–178. https://doi.org/10.1016/0165-5728(90)90088-5.

27. D'Amelio, F., Eng, L.F., and Gibbs, M.A. (1990). Glutamine synthetase immunoreactivity is present in oligodendroglia of various regions of the central nervous system. GLIA (New York, N. Y.) *3*, 335–341. https://doi.org/10.1002/glia.440030504.

28. Xin, W., Mironova, Y.A., Shen, H., Marino, R.A.M., Waisman, A., Lamers, W.H., Bergles, D.E., and Bonci, A. (2019). Oligodendrocytes Support Neuronal Glutamatergic Transmission via Expression of Glutamine Synthetase. Cell Rep. *27*, 2262–2271.e5. https://doi.org/10.1016/j.celrep.2019.04.094.

29. Zhang, J.S., Liu, Y., Xu, J.H., Yang, P.B., Xiao, X.L., Chen, X.L., Tian, Y.M., and Zhang, J.F. (2008). [Neurons in the corpus callosum of rats: expression of Cav2.2 and their connection]. Zhong Nan Da Xue Xue Bao Yi Xue Ban *33*, 99–102.

30. Wang, H., Xu, L., Lai, C., Hou, K., Chen, J., Guo, Y., Sambangi, A., Swaminathan, S., Xie, C., Wu, Z., and Chen, G. (2021). Region-specific distribution of Olig2-expressing astrocytes in adult mouse brain and spinal cord. Mol. Brain *14*, 36.

31. Zhou, Q., Wang, S., and Anderson, D.J. (2000). Identification of a Novel Family of Oligodendrocyte Lineage-Specific Basic Helix–Loop–Helix Transcription Factors. Neuron (Camb., Mass.) *25*, 331–343. https://doi.org/10.1016/S0896-6273(00)80898-3.

32. Lu, Q.R., Yuk, D., Alberta, J.A., Zhu, Z., Pawlitzky, I., Chan, J., McMahon, A.P., Stiles, C.D., and Rowitch, D.H. (2000). Sonic hedgehog-regulated oligodendrocyte lineage genes encoding bHLH proteins in the mammalian central nervous system. Neuron (Camb., Mass.) *25*, 317–329. https://doi.org/10.1016/S0896-6273(00)80897-1.

33. von Jonquieres, G., Fröhlich, D., Klugmann, C.B., Wen, X., Harasta, A.E., Ramkumar, R., Spencer, Z.H.T., Housley, G.D., and Klugmann, M. (2016). Recombinant human myelin-associated glycoprotein promoter drives selective AAV-mediated transgene expression in oligodendrocytes. Front. Mol. Neurosci. *9*, 13. https://doi.org/10.3389/fnmol.2016.00013.

34. von Jonquieres, G., Mersmann, N., Klugmann, C.B., Harasta, A.E., Lutz, B., Teahan, O., Housley, G.D., Fröhlich, D., Krämer-Albers, E.M., and Klugmann, M. (2013). Glial Promoter Selectivity following AAV-Delivery to the Immature Brain. PLoS One *8*, e65646. https://doi.org/10.1371/JOURNAL.PONE.0065646.

35. Duan, W., Zhang, Y.P., Hou, Z., Huang, C., Zhu, H., Zhang, C.Q., and Yin, Q. (2016). Novel Insights into NeuN: from Neuronal Marker to Splicing Regulator. Mol. Neurobiol. *53*, 1637–1647. https://doi.org/10.1007/S12035-015-9122-5/FIGURES/1.

36. Hordeaux, J., Yuan, Y., Clark, P.M., Wang, Q., Martino, R.A., Sims, J.J., Bell, P., Raymond, A., Stanford, W.L., and Wilson, J.M. (2019). The GPI-Linked Protein LY6A Drives AAV-PHP.B Transport across the Blood-Brain Barrier. Mol. Ther. *27*, 912–921. https://doi.org/10.1016/J.YMTHE.2019.02.013.

37. Batista, A.R., King, O.D., Reardon, C.P., Davis, C., Shankaracharya, P.,V., Philip, V., Gray-Edwards, H., Aronin, N., Lutz, C., Landers, J., and Sena-Esteves, M. (2020). Ly6a Differential Expression in Blood-Brain Barrier Is Responsible for Strain Specific Central Nervous System Transduction Profile of AAV-PHP.B. Hum. Gene Ther. *31*, 90–102. https://doi.org/10.1089/hum.2019.186.

38. Huang, Q., Chan, K.Y., Tobey, I.G., Chan, Y.A., Poterba, T., Boutros, C.L., Balazs, A. B., Daneman, R., Bloom, J.M., Seed, C., and Deverman, B.E. (2019). Delivering genes across the blood-brain barrier: LY6A, a novel cellular receptor for AAV-PHP.B capsids. PLoS One *14*, e0225206–e0225217. https://doi.org/10.1371/journal.pone.0225206.

39. Bennett, J., Basivireddy, J., Kollar, A., Biron, K.E., Reickmann, P., Jefferies, W.A., and McQuaid, S. (2010). Blood–brain barrier disruption and enhanced vascular permeability in the multiple sclerosis model EAE. J. Neuroimmunol. *229*, 180–191. https://doi.org/10.1016/J.JNEUROIM.2010.08.011.

40. Ayzenberg, I., Schlevogt, S., Metzdorf, J., Stahlke, S., Pedreitturia, X., Hunfeld, A., Couillard-Despres, S., and Kleiter, I. (2015). Analysis of Neurogenesis during Experimental Autoimmune Encephalomyelitis Reveals Pitfalls of Bioluminescence Imaging. PLoS One *10*, e0118550. https://doi.org/10.1371/JOURNAL.PONE.0118550.

41. Goertsen, D., Flytzanis, N.C., Goeden, N., Chuapoco, M.R., Cummins, A., Chen, Y., Fan, Y., Zhang, Q., Sharma, J., Duan, Y., et al. (2022). AAV capsid variants with

brain-wide transgene expression and decreased liver targeting after intravenous delivery in mouse and marmoset. Nat. Neurosci. *25*, 106–115. https://doi.org/10.1038/s41593-021-00969-4.

42. Handyside, B., Ismail, A.M., Zhang, L., Yates, B., Xie, L., Sihn, C.R., Murphy, R., Bouwman, T., Kim, C.K., De Angelis, R., et al. (2022). Vector genome loss and epigenetic modifications mediate decline in transgene expression of AAV5 vectors produced in mammalian and insect cells. Mol. Ther. *30*, 3570–3586. https://doi.org/10.1016/j.ymthe.2022.11.001.

43. Gonzalez-Sandoval, A., Pekrun, K., Tsuji, S., Zhang, F., Hung, K.L., Chang, H.Y., and Kay, M.A. (2023). The AAV capsid can influence the epigenetic marking of rAAV delivered episomal genomes in a species dependent manner. Nat. Commun. *14*, 2448. https://doi.org/10.1038/s41467-023-38106-3.

44. Zhu, Y., Wang, G.Z., Cingöz, O., and Goff, S.P. (2018). NP220 mediates silencing of unintegrated retroviral DNA. Nature *564*, 278–282. https://doi.org/10.1038/S41586-018-0750-6.

45. Nuo, M.T., Yuan, J.L., Yang, W.L., Gao, X.Y., He, N., Liang, H., Cang, M., and Liu, D. J. (2016). Promoter methylation and histone modifications affect the expression of the exogenous DsRed gene in transgenic goats. Genet. Mol. Res. *15*. https://doi.org/10.4238/gmr.15038560.

46. Cabrera, A., Edelstein, H.I., Glykofrydis, F., Love, K.S., Palacios, S., Tycko, J., Zhang, M., Lensch, S., Shields, C.E., Livingston, M., et al. (2022). The sound of silence: Transgene silencing in mammalian cell engineering. Cell Syst. *13*, 950–973. https://doi.org/10.1016/J.CELS.2022.11.005.

47. Darras, B., Mercuri, E., Strauss, K., Day, J., Chien, Y.-H., Masson, R., Wigderson, M., Alecu, I., Ballarini, N., Mehl, L., et al. (2023). Intravenous and Intrathecal Onasemnogene Abeparvovec Gene Therapy in Symptomatic and Presymptomatic Spinal Muscular Atrophy: Long-Term Follow-Up Study (S34.002). Neurology *100*, 2439. https://doi.org/10.1212/WNL.0000000000202555.

48. Mendell, J., Shell, R., Lehman, K., McColly, M., Lowes, L., Alfano, L., Miller, N., Iammarino, M., Church, K., Ogrinc, F., et al. (2020). SMA – THERAPY: P.261 Long-term follow-up of onasemnogene abeparvovec gene therapy in spinal muscular atrophy type 1 (SMA1). Neuromuscul. Disord. *30*, S122–S123. https://doi.org/10.1016/J.NMD.2020.08.260.

49. Finneran, D.J., Njoku, I.P., Flores-Pazarin, D., Ranabothu, M.R., Nash, K.R., Morgan, D., and Gordon, M.N. (2021). Toward Development of Neuron Specific Transduction After Systemic Delivery of Viral Vectors. Front. Neurol. *12*, 685802. https://doi.org/10.3389/fneur.2021.685802.

50. Chand, D., Mohr, F., McMillan, H., Tukov, F.F., Montgomery, K., Kleyn, A., Sun, R., Tauscher-Wisniewski, S., Kaufmann, P., and Kullak-Ublick, G. (2021). Hepatotoxicity following administration of onasemnogene abeparvovec (AVXS-101) for the treatment of spinal muscular atrophy. J. Hepatol. *74*, 560–566. https://doi.org/10.1016/J.JHEP.2020.11.001.

51. Carotti, S., Morini, S., Corradini, S.G., Burza, M.A., Molinaro, A., Carpino, G., Merli, M., De Santis, A., Muda, A.O., Rossi, M., et al. (2008). Glial fibrillary acidic protein as an early marker of hepatic stellate cell activation in chronic and posttransplant recurrent hepatitis C. Liver Transpl. *14*, 806–814. https://doi.org/10.1002/LT.21436.

52. Kim, J.W., and Kim, Y.J. (2024). The evidence-based multifaceted roles of hepatic stellate cells in liver diseases: A concise review. Life Sci. *344*, 122547. https://doi.org/10.1016/j.lfs.2024.122547.

53. Lim, M.C.C., Maubach, G., and Zhuo, L. (2008). Glial Fibrillary Acidic Protein Splice Variants in Hepatic Stellate Cells - Expression and Regulation. Mol. Cells *25*, 376–384. https://doi.org/10.1016/s1016-8478(23)17595-9.

54. Nieuwenhuis, B., Haenzi, B., Hilton, S., Carnicer-Lombarte, A., Hobo, B., Verhaagen, J., and Fawcett, J.W. (2021). Optimization of adeno-associated viral vector-mediated transduction of the corticospinal tract: comparison of four promoters. Gene Ther. *28*, 56–74. https://doi.org/10.1038/s41434-020-0169-1.

55. Chai, S., Wakefield, L., Norgard, M., Li, B., Enicks, D., Marks, D.L., and Grompe, M. (2023). Strong ubiquitous micro-promoters for recombinant adeno-associated viral vectors. Mol. Ther. Methods Clin. Dev. *29*, 504–512. https://doi.org/10.1016/j.omtm.2023.05.013.

56. Pignataro, D., Sucunza, D., Vanrell, L., Lopez-Franco, E., Dopeso-Reyes, I.G., Vales, A., Hommel, M., Rico, A.J., Lanciego, J.L., and Gonzalez-Aseguinolaza, G. (2017). Adeno-associated viral vectors serotype 8 for cell-specific delivery of therapeutic genes in the central nervous system. Front. Neuroanat. *11*, 2. https://doi.org/10.3389/FNANA.2017.00002/FULL.

57. Shevtsova, Z., Malik, J.M.I., Michel, U., Bähr, M., and Kügler, S. (2005). Promoters and serotypes: targeting of adeno-associated virus vectors for gene transfer in the rat central nervous system in vitro and in vivo. Exp. Physiol. *90*, 53–59. https://doi.org/10.1113/EXPPHYSIOL.2004.028159.

58. Hilton, B.J., Anenberg, E., Harrison, T.C., Boyd, J.D., Murphy, T.H., and Tetzlaff, W. (2016). Re-Establishment of Cortical Motor Output Maps and Spontaneous Functional Recovery via Spared Dorsolaterally Projecting Corticospinal Neurons after Dorsal Column Spinal Cord Injury in Adult Mice. J. Neurosci. *36*, 4080–4092. https://doi.org/10.1523/JNEUROSCI.3386-15.2016.

59. Hioki, H., Kameda, H., Nakamura, H., Okunomiya, T., Ohira, K., Nakamura, K., Kuroda, M., Furuta, T., and Kaneko, T. (2007). Efficient gene transduction of neurons by lentivirus with enhanced neuron-specific promoters. Gene Ther. *14*, 872–882. https://doi.org/10.1038/sj.gt.3302924.

60. Thiel, G., Greengard, P., and Südhof, T.C. (1991). Characterization of tissue-specific transcription by the human synapsin I gene promoter. Proc. Natl. Acad. Sci. USA *88*, 3431–3435. https://doi.org/10.1073/PNAS.88.8.3431.

61. McLean, J.R., Smith, G.A., Rocha, E.M., Hayes, M.A., Beagan, J.A., Hallett, P.J., and Isacson, O. (2014). Widespread neuron-specific transgene expression in brain and spinal cord following synapsin promoter-driven AAV9 neonatal intracerebroventricular injection. Neurosci. Lett. *576*, 73–78. https://doi.org/10.1016/J.NEULET.2014.05.044.

62. Kügler, S., Kilic, E., and Bähr, M. (2003). Human synapsin 1 gene promoter confers highly neuron-specific long-term transgene expression from an adenoviral vector in the adult rat brain depending on the transduced area. Gene Ther. *10*, 337–347. https://doi.org/10.1038/SJ.GT.3301905.

63. Gennaro, M., Mattiello, A., Mazziotti, R., Antonelli, C., Gherardini, L., Guzzetta, A., Berardi, N., Cioni, G., and Pizzorusso, T. (2017). Focal stroke in the developing rat motor cortex induces age- and experience-dependent maladaptive plasticity of corticospinal system. Front. Neural Circuits *11*, 47. https://doi.org/10.3389/FNCIR.2017.00047/FULL.

64. Watakabe, A., Ohtsuka, M., Kinoshita, M., Takaji, M., Isa, K., Mizukami, H., Ozawa, K., Isa, T., and Yamamori, T. (2015). Comparative analyses of adeno-associated viral vector serotypes 1, 2, 5, 8 and 9 in marmoset, mouse and macaque cerebral cortex. Neurosci. Res. *93*, 144–157. https://doi.org/10.1016/J.NEURES.2014.09.002.

65. Watakabe, A., Sadakane, O., Hata, K., Ohtsuka, M., Takaji, M., and Yamamori, T. (2017). Application of viral vectors to the study of neural connectivities and neural circuits in the marmoset brain. Dev. Neurobiol. *77*, 354–372. https://doi.org/10.1002/DNEU.22459.

66. Jiang, R., Diaz-Castro, B., Looger, L.L., and Khakh, B.S. (2016). Dysfunctional Calcium and Glutamate Signaling in Striatal Astrocytes from Huntington's Disease Model Mice. J. Neurosci. *36*, 3453–3470. https://doi.org/10.1523/JNEUROSCI.3693-15.2016.

67. Nagai, J., Rajbhandari, A.K., Gangwani, M.R., Hachisuka, A., Coppola, G., Masmanidis, S.C., Fanselow, M.S., and Khakh, B.S. (2019). Hyperactivity with Disrupted Attention by Activation of an Astrocyte Synaptogenic Cue. Cell *177*, 1280–1292.e20. https://doi.org/10.1016/J.CELL.2019.03.019/ATTACHMENT/43F2B535-2C38-4526-8664-079CAFF3C20D/MMC2.XLSX.

68. Yu, X., Taylor, A.M.W., Nagai, J., Golshani, P., Evans, C.J., Coppola, G., and Khakh, B.S. (2018). Reducing astrocyte calcium signaling in vivo alters striatal microcircuits and causes repetitive behavior. Neuron (Camb., Mass.) *99*, 1170–1187.e9. https://doi.org/10.1016/J.NEURON.2018.08.015.

69. Octeau, J.C., Chai, H., Jiang, R., Bonanno, S.L., Martin, K.C., and Khakh, B.S. (2018). An optical neuron-astrocyte proximity assay at synaptic distance scales. Neuron (Camb., Mass.) *98*, 49–66.e9. https://doi.org/10.1016/J.NEURON.2018.03.003.

70. Xie, Y., Wang, T., Sun, G.Y., and Ding, S. (2010). Specific Disruption of Astrocytic Ca2+ Signaling Pathway in vivo by Adeno-Associated Viral Transduction. Neuroscience *170*, 992–1003. https://doi.org/10.1016/J.NEUROSCIENCE.2010.08.034.

71. Poskanzer, K.E., and Yuste, R. (2016). Astrocytes regulate cortical state switching in vivo. Proc. Natl. Acad. Sci. USA *113*, E2675–E2684. https://doi.org/10.1073/PNAS.1520759113/-/DCSUPPLEMENTAL.

72. Brenner, M., Kisseberth, W.C., Su, Y., Besnard, F., and Messing, A. (1994). GFAP promoter directs astrocyte-specific expression in transgenic mice. J. Neurosci. *14*, 1030–1037. https://doi.org/10.1523/JNEUROSCI.14-03-01030.1994.

73. Aquino, D.A., Chiu, F.-C., Brosnan, C.F., and Norton, W.T. (1988). Glial Fibrillary Acidic Protein Increases in the Spinal Cord of Lewis Rats with Acute Experimental Autoimmune Encephalomyelitis. J. Neurochem. *51*, 1085–1096. https://doi.org/10.1111/J.1471-4159.1988.TB03072.X.

74. Aquino, D.A., Shafit-Zagardo, B., Brosnan, C.F., and Norton, W.T. (1990). Expression of glial fibrillary acidic protein and neurofilament mRNA in gliosis induced by experimental autoimmune encephalomyelitis. J. Neurochem. *54*, 1398–1404. https://doi.org/10.1111/J.1471-4159.1990.TB01975.X.

75. Küspert, M., Hammer, A., Bösl, M.R., and Wegner, M. (2011). Olig2 regulates Sox10 expression in oligodendrocyte precursors through an evolutionary conserved distal enhancer. Nucleic Acids Res. *39*, 1280–1293. https://doi.org/10.1093/nar/gkq951.

76. Dawson, M.R.L., Polito, A., Levine, J.M., and Reynolds, R. (2003). NG2-expressing glial progenitor cells: an abundant and widespread population of cycling cells in the adult rat CNS. Mol. Cell. Neurosci. *24*, 476–488. https://doi.org/10.1016/S1044-7431(03)00210-0.

77. Fernandez-Castaneda, A., and Gaultier, A. (2016). Adult oligodendrocyte progenitor cells – Multifaceted regulators of the CNS in health and disease. Brain Behav. Immun. *57*, 1–7. https://doi.org/10.1016/J.BBI.2016.01.005.

78. Pietersz, K.L., Plessis, F.D., Pouw, S.M., Liefhebber, J.M., van Deventer, S.J., Martens, G.J.M., Konstantinova, P.S., and Blits, B. (2021). PhP.B Enhanced Adeno-Associated Virus Mediated-Expression Following Systemic Delivery or Direct Brain Administration. Front. Bioeng. Biotechnol. *9*, 679483. https://doi.org/10.3389/FBIOE.2021.679483.

79. Samaranch, L., Salegio, E.A., San Sebastian, W., Kells, A.P., Foust, K.D., Bringas, J.R., Lamarre, C., Forsayeth, J., Kaspar, B.K., and Bankiewicz, K.S. (2012). Adeno-associated virus serotype 9 transduction in the central nervous system of nonhuman primates. Hum. Gene Ther. *23*, 382–389. https://doi.org/10.1089/hum.2011.200.

80. Powell, S.K., Khan, N., Parker, C.L., Samulski, R.J., Matsushima, G., Gray, S.J., and McCown, T.J. (2016). Characterization of a novel adeno-associated viral vector with preferential oligodendrocyte tropism. Gene Ther. *23*, 807–814. https://doi.org/10.1038/gt.2016.62.

81. De Leeuw, B., Su, M., Ter Horst, M., Iwata, S., Rodijk, M., Hoeben, R.C., Messing, A., Smitt, P.S., and Brenner, M. (2006). Increased glia-specific transgene expression with glial fibrillary acidic protein promoters containing multiple enhancer elements. J. Neurosci. Res. *83*, 744–753. https://doi.org/10.1002/jnr.20776.

82. Kügler, S., Lingor, P., Schöll, U., Zolotukhin, S., and Bähr, M. (2003). Differential transgene expression in brain cells in vivo and in vitro from AAV-2 vectors with small transcriptional control units. Virology (New York, N. Y.) *311*, 89–95. https://doi.org/10.1016/S0042-6822(03)00162-4.

83. Korecka, J., Schouten, M., Eggers, R., Ulusoy, A., Bossers, K., and Verhaage, J. (2011). Comparison of AAV Serotypes for Gene Delivery to Dopaminergic Neurons in the Substantia Nigra. Viral Gene Therapy *2*, 1–22. https://doi.org/10.5772/18939.

84. Pietersz, K.L., Nijhuis, P.J.H., Klunder, M.H.M., van den Herik, J., Hobo, B., de Winter, F., and Verhaagen, J. (2024). Author Spotlight: Advancing Gene Therapy with High-Yield AAV Vectors Through HEK293 Suspension Cell Cultivation. J. Vis. Exp. *2024*, e66532. https://doi.org/10.3791/66532.

OMTM, Volume *33*

Supplemental information

Developing a minimally invasive gene therapy for multiple sclerosis

Paul J.H. Nijhuis, Maurits Romijn, Roy Honing, Giselle van Zon, Inge Huitinga, Fred de Winter, and Joost Verhaagen

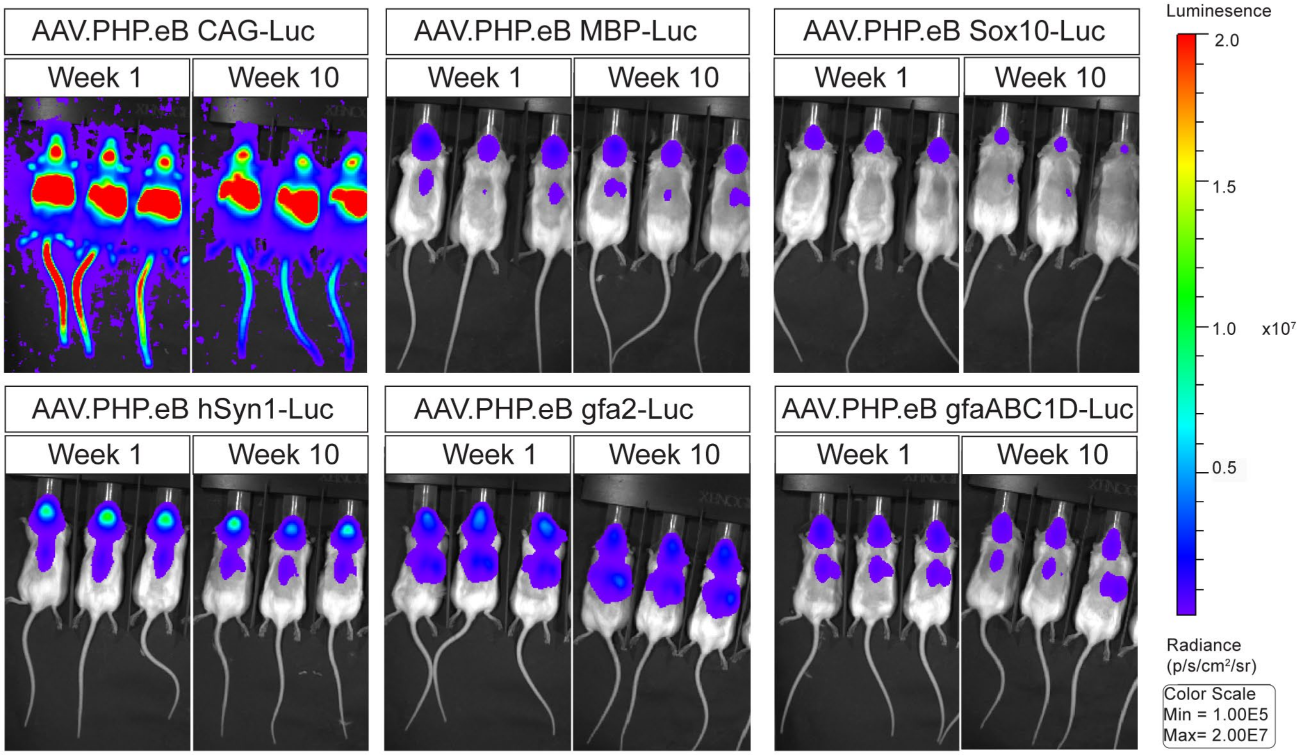

Figure S1. *In vivo* bioluminescence images after one and ten weeks *post*-intravenous AAV administration. Exemplary images of the whole-body bioluminescence in healthy mice after one and ten weeks luciferase expression directed by the general CAG promoter and the CNS directed neural promoters.

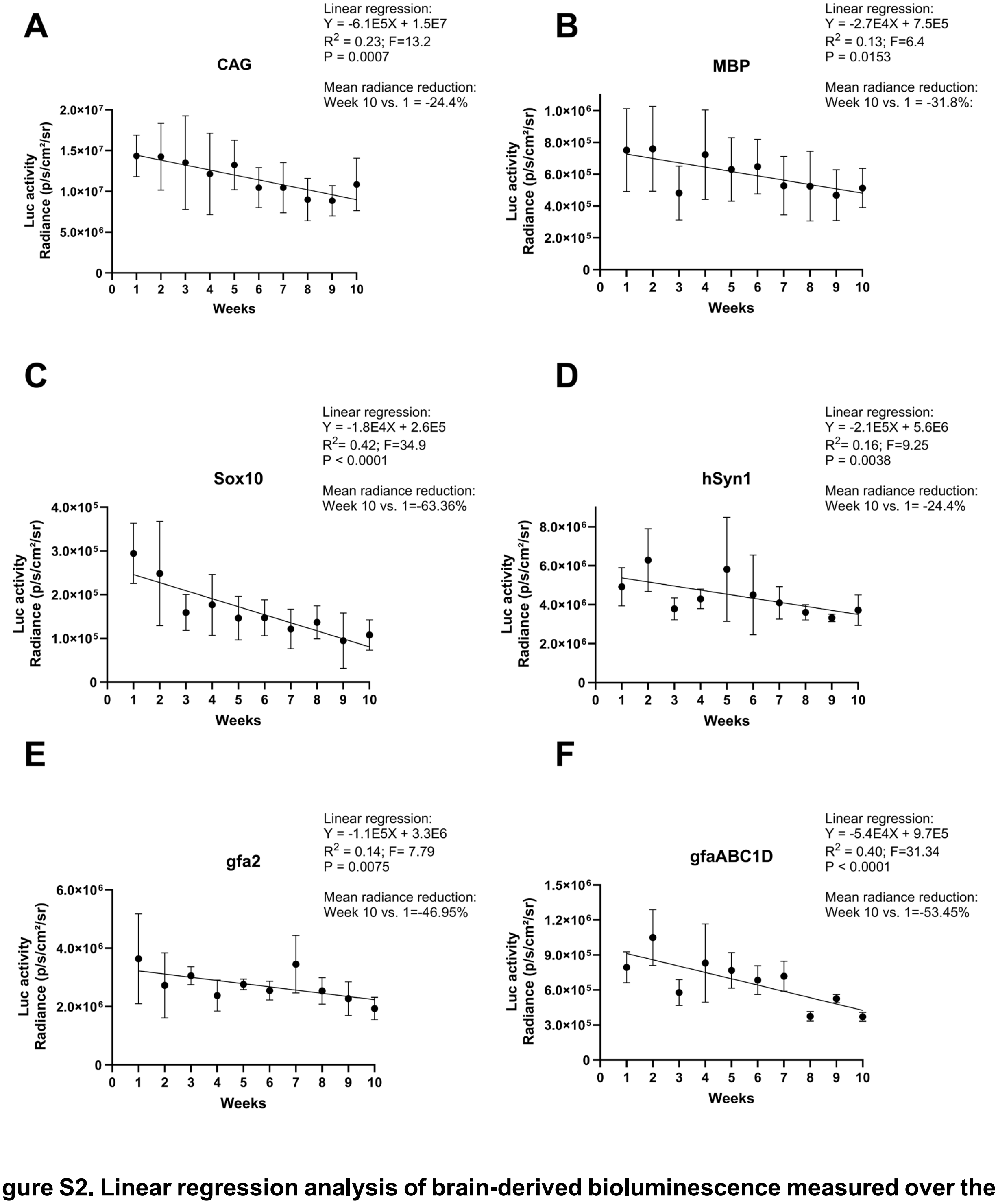

Figure S2. Linear regression analysis of brain-derived bioluminescence measured over the course of ten weeks A-F. Linear regression analysis of all promoters revealed a gradual but significant downwards trend of bioluminescence signal during ten subsequent weekly measurements. All promoters showed a significant decrease in bioluminescence signal at week 10 versus week 1. n=5 per group,57Bl/6N (B6N-Tyrc-Brd/BrdCrCrl), results represent mean (SD).

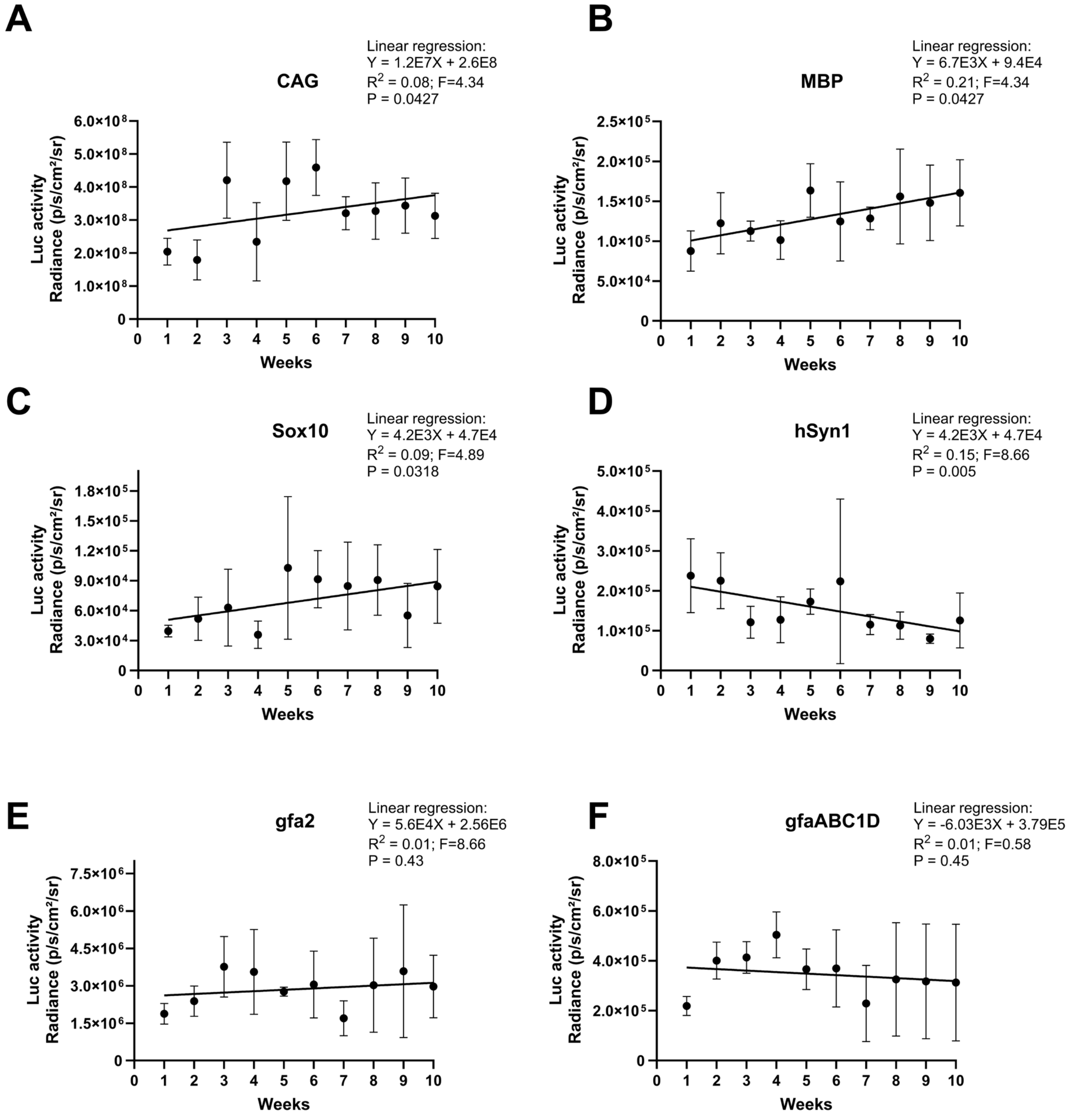

Figure S3. Linear regression analysis of liver-associated bioluminescence measured over the course of ten weeks A-F. Linear regression analysis revealed a gradual but significant upward correlation of the liver-associated bioluminescence signal over the ten weeks measurements period for the CAG, MBP and Sox10 promoters. Liver bioluminescence significantly decreased over the course of the experiment for the hSyn1 promoter. Liver bioluminescence remained stable for gfa2 and gfaABC1D. n=5 per group, C57Bl/6N (B6N-Tyrc-Brd/BrdCrCrl), results represent the mean (SD).

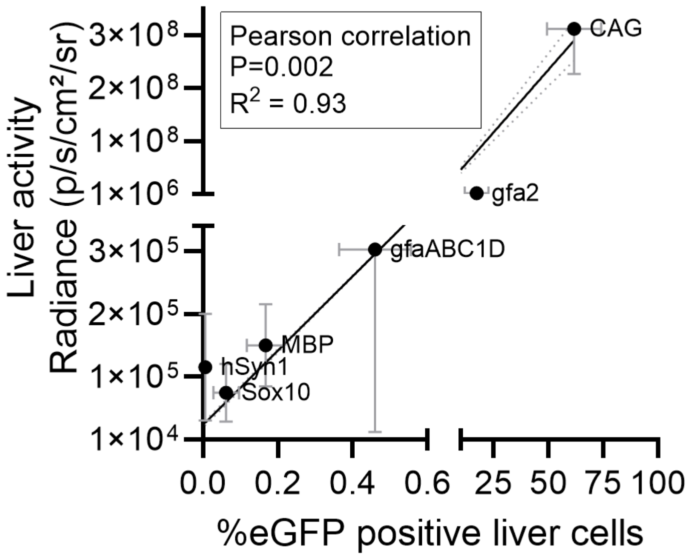

Figure S4. Liver *in vivo* bioluminescence at week ten correlated positively with the proportion of eGFP labeled liver cells. Measured liver bioluminescence was plotted against the percentage of eGFP-positive liver cells. Data represented as mean (SD) in for both axis. Pearson correlation demonstrates a significant correlation (P=0.002; R^2=0.93) between the *in vivo* readout with luciferase and eGFP immunohistochemical analysis of liver expression.

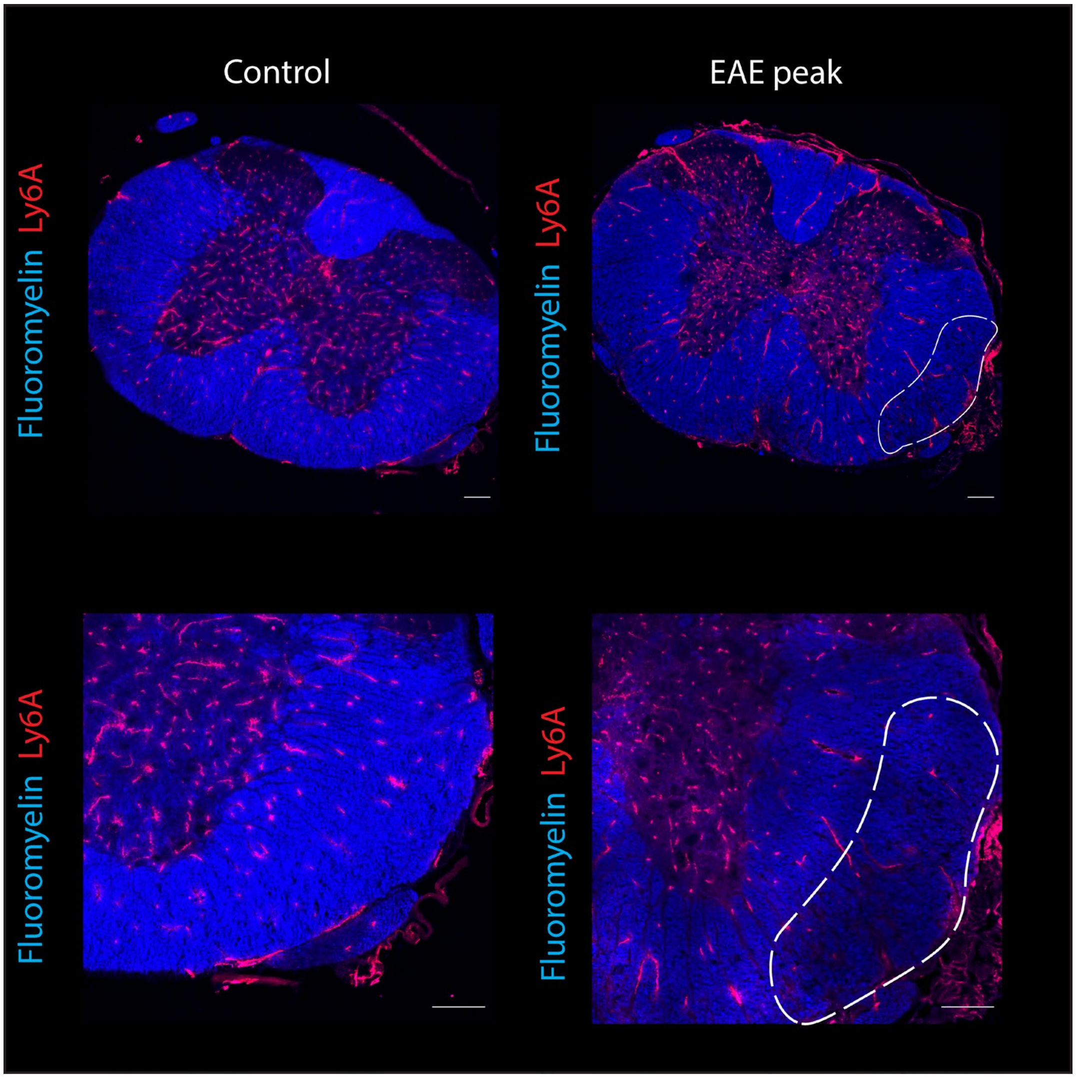

Figure S5. Presence of the endothelial Ly6A receptor in the healthy spinal cord and at the chronic phase of EAE. A. Healthy spinal cord tile scan (Z-stack) showing presence of myelin (fluoromyelin, blue) and the Ly6A receptor (red) in mouse spinal cord grey and white matter tissue. **B**. Higher magnification (x20, dry) of showing presence of myelin (fluoromyelin, blue) and the Ly6A receptor (magenta) in mouse spinal cord grey and white matter tissue. **C**. EAE affected spinal cord at 18 days post MOG_{35-55} immunization (chronic EAE phase). Stained for fluoromyelin and Ly6A reveal myelin damage and presence of the Ly6A receptor distal, proximal and within the EAE-lesioned spinal cord. **D**. Higher magnification (x20, dry) of the EAE affected spinal cord at 18 days post MOG_{35-55} immunization (chronic EAE phase) reveals myelin damage and sustained presence of the Ly6A receptor distal, proximal and within the EAE lesioned spinal cord.

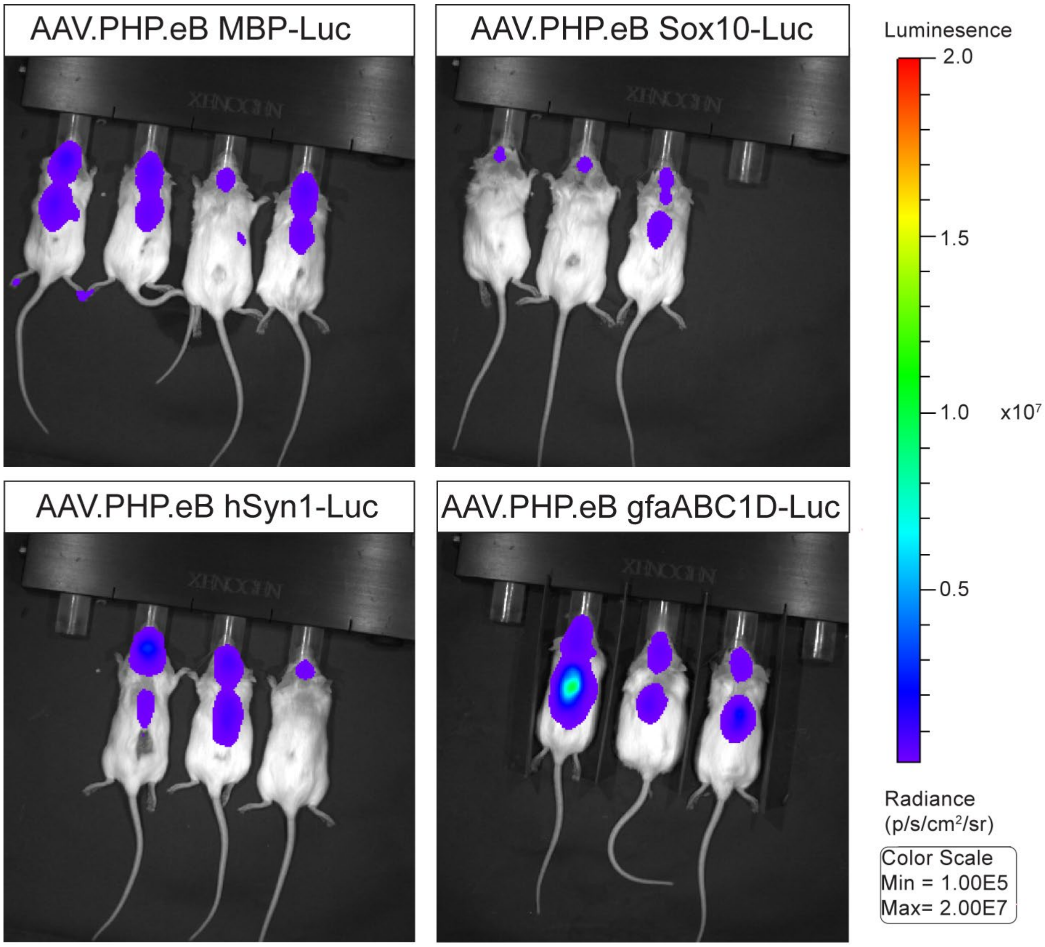

Figure S6. *In vivo* bioluminescence images of EAE mice, four weeks *post*-intravenous AAV administration. Mice were administered AAV.PHP.eB five days after EAE-induction to monitor the expression of luciferase over the EAE disease course. After four weeks the transgene was still highly expressed during the chronic phase of EAE.

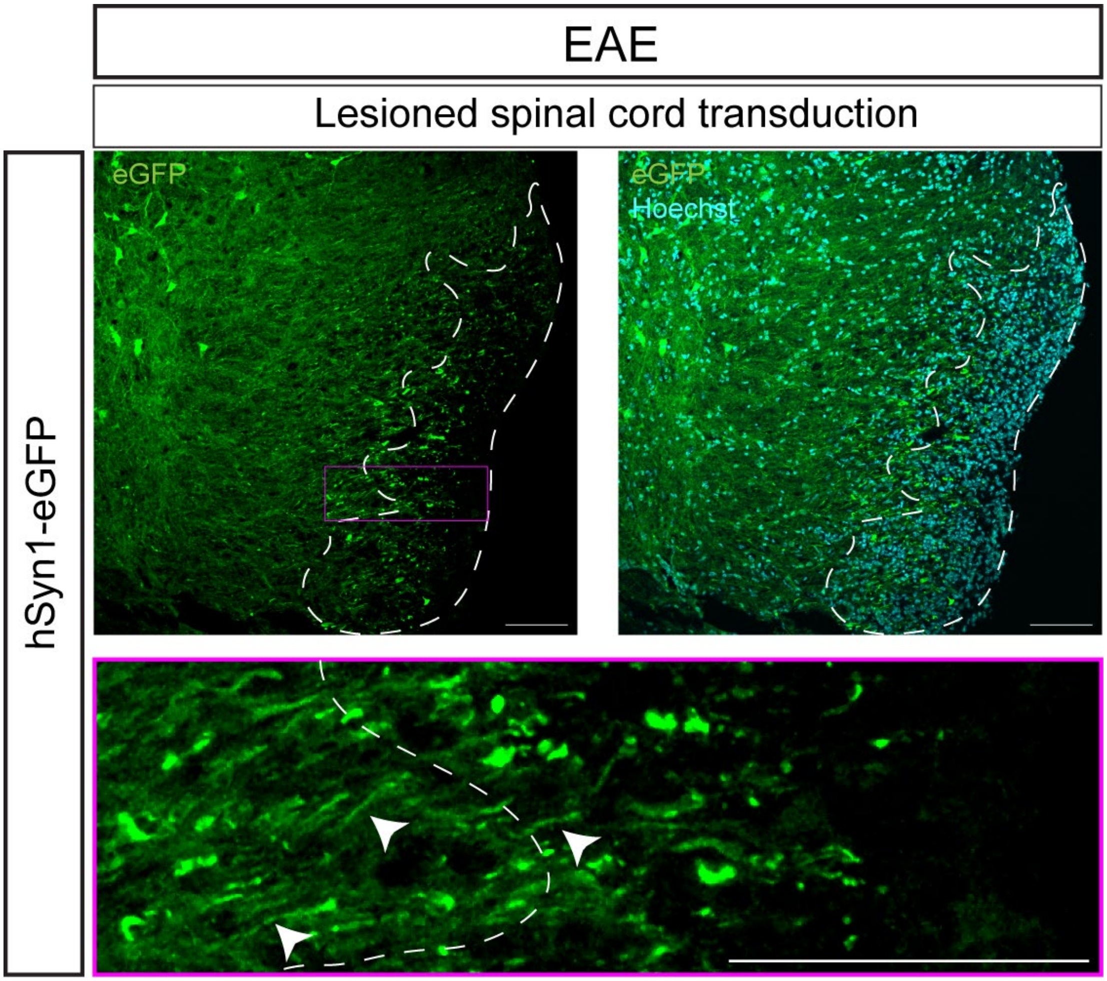

Figure S7. eGFP-labeled axons are present within the EAE lesion but show signs of axonal degeneration at the lesion edge, close to the rim of the tissue. In the spinal cord after four weeks of expression, eGFP-labeled axons can be seen crossing the lesion rim (white arrowheads). Loss and of eGFP-labeled axons near the rim of the tissue suggests axonal degeneration and fragmentation as a result of inflammation. Scale bar = 100 μm.
